# SARS-CoV-2 main protease cleaves MAGED2 to antagonize host antiviral defense

**DOI:** 10.1128/mbio.01373-23

**Published:** 2023-07-13

**Authors:** Xiaohui Ju, Ziqiao Wang, Pengcheng Wang, Wenlin Ren, Yanying Yu, Yin Yu, Bin Yuan, Jingwei Song, Xiaochun Zhang, Yu Zhang, Chang Xu, Boxue Tian, Yi Shi, Rong Zhang, Qiang Ding

**Affiliations:** 1 School of Medicine, Tsinghua University, Beijing, China; 2 Key Laboratory of Medical Molecular Virology (MOE/NHC/CAMS), School of Basic Medical Sciences, Shanghai Medical College, Biosafety Level 3 Laboratory, Fudan University, Shanghai, China; 3 CAS Key Laboratory of Pathogenic Microbiology and Immunology, Institute of Microbiology, Chinese Academy of Sciences, Beijing, China; 4 School of Pharmaceutical Sciences, Tsinghua University, Beijing, China; Virginia Polytechnic Institute and State University, Blacksburg, Virginia, USA

**Keywords:** SARS-CoV-2, main protease, MAGED2, cleavage, viral replication, nucleocapsid protein

## Abstract

Severe acute respiratory syndrome coronavirus 2 (SARS-CoV-2) is the agent causing the global pandemic of COVID-19. SARS-CoV-2 genome encodes a main protease (nsp5, also called Mpro) and a papain-like protease (nsp3, also called PLpro), which are responsible for processing viral polyproteins to assemble a functional replicase complex. In this study, we found that Mpro of SARS-CoV-2 can cleave human MAGED2 and other mammalian orthologs at Gln-263. Moreover, SARS-CoV and MERS-CoV Mpro can also cleave human MAGED2, suggesting MAGED2 cleavage by Mpro is an evolutionarily conserved mechanism of coronavirus infection in mammals. Intriguingly, Mpro from Beta variant cleaves MAGED2 more efficiently than wild type, but Omicron Mpro is opposite. Further studies show that MAGED2 inhibits SARS-CoV-2 infection at viral replication step. Mechanistically, MAGED2 is associated with SARS-CoV-2 nucleocapsid protein through its N-terminal region in an RNA-dependent manner, and this disrupts the interaction between SARS-CoV-2 nucleocapsid protein and viral genome, thus inhibiting viral replication. When MAGED2 is cleaved by Mpro, the N-terminal of MAGED2 will translocate into the nucleus, and the truncated MAGED2 is unable to suppress SARS-CoV-2 replication. This work not only discovers the antiviral function of MAGED2 but also provides new insights into how SARS-CoV-2 Mpro antagonizes host antiviral response.

**IMPORTANCE:**

Host factors that restrict severe acute respiratory syndrome coronavirus 2 (SARS-CoV-2) infection remain elusive. Here, we found that MAGED2 can be cleaved by SARS-CoV-2 main protease (Mpro) at Gln-263. SARS-CoV and MERS-CoV Mpro can also cleave MAGED2, and MAGED2 from multiple species can be cleaved by SARS-CoV-2 Mpro. Mpro from Beta variant cleaves MAGED2 more efficiently efficiently than wild type, but Omicron is the opposite. MAGED2 depletion enhances SARS-CoV-2 infection, suggesting its inhibitory role in SARS-CoV-2 infection. Mechanistically, MAGED2 restricts SARS-CoV-2 replication by disrupting the interaction between nucleocapsid and viral genomes. When MAGED2 is cleaved, its N-terminal will translocate into the nucleus. In this way, Mpro relieves MAGED2' inhibition on viral replication. This study improves our understanding of complex viral-host interaction and provides novel targets to treat SARS-CoV-2 infection.

## INTRODUCTION

The pandemic of coronavirus disease 2019 (COVID-19) caused by severe acute respiratory syndrome coronavirus 2 (SARS-CoV-2) damages people’s health severely. Even though vaccines and direct-acting antivirals against SARS-CoV-2 are available, the emergence of new variants of the SARS-CoV-2 (Alpha, Beta, Gamma, Delta, and Omicron) challenges their efficacy (1, 2). SARS-CoV-2 is an enveloped, positive-sense RNA virus containing a 30-kb single-stranded RNA genome (3, 4). Viral genome encodes two large non-structural proteins (pp1a and pp1ab), which are processed by nsp3 (PLpro) and nsp5 (Mpro) into individual proteins to form the viral replication and transcription complex (5). Structural proteins, including spike (S), envelope (E), membrane (M), and nucleocapsid (N) proteins, are translated from subgenomic RNAs (6). The accessory proteins, encoded by ORF3a, ORF3b, ORF6, ORF7a, ORF7b, ORF8, ORF9b, and ORF10 genes, are not directly involved in viral replication but repress the host innate immune response (7 - 11). Besides its function in cleavage of viral large non-structure proteins, accumulating evidences have found that Mpro can cleave host proteins such as TAB1, NLRP12, RIG-I, NEMO, and RNF20 to antagonize host antiviral defense (12 - 15).

Melanoma-associated antigen D2 (MAGED2) is ubiquitously expressed in normal adult tissues (16) and localizes in both cytoplasm and nucleus (17). MAGED2 has been reported to regulate DNA damage response and maintain genomic stability (18). MAGED2 can inhibit tumor necrosis factor (TNF)-related apoptosis-inducing ligand (TRAIL)-induced apoptosis (19). Besides, MAGED2 protects Na-K-Cl cotransporter (NKCC2) and Na-Cl cotransporter (NCC), which are important regulators of salt reabsorption, from Hsp40-mediated degradation (20). However, the biological role of MAGED2 in virus infection has been less characterized.

Here, we found that human MAGED2 can be cleaved by SARS-CoV-2, SARS-CoV, and MERS-CoV Mpro at Gln-263, and MAGED2 orthologs from monkey, cat, bat, pangolin, and mouse can be cleaved by SARS-CoV-2 Mpro. Genetic ablation of MAGED2 increased SARS-CoV-2 infection, suggesting that MAGED2 acts as an intrinsic restriction factor of SARS-CoV-2 infection. Mechanistic studies showed that MAGED2 can interact with SARS-CoV-2 N protein through its N-terminal region in an RNA-dependent manner, and this can disrupt the interaction between N protein and SARS-CoV-2 genomic RNA. Upon cleaved by Mpro at Gln-263, the N-terminal region of MAGED2 will translocate into the nucleus, thus unable to inhibit SARS-CoV-2 replication. This work provides novel insights into viral-host interaction and discovers a new function of MAGED2 with antiviral activity against SARS-CoV-2 infection.

## RESULTS

### SARS-CoV-2 Mpro cleaves MAGED2 at Gln-263

**Fig 1:**
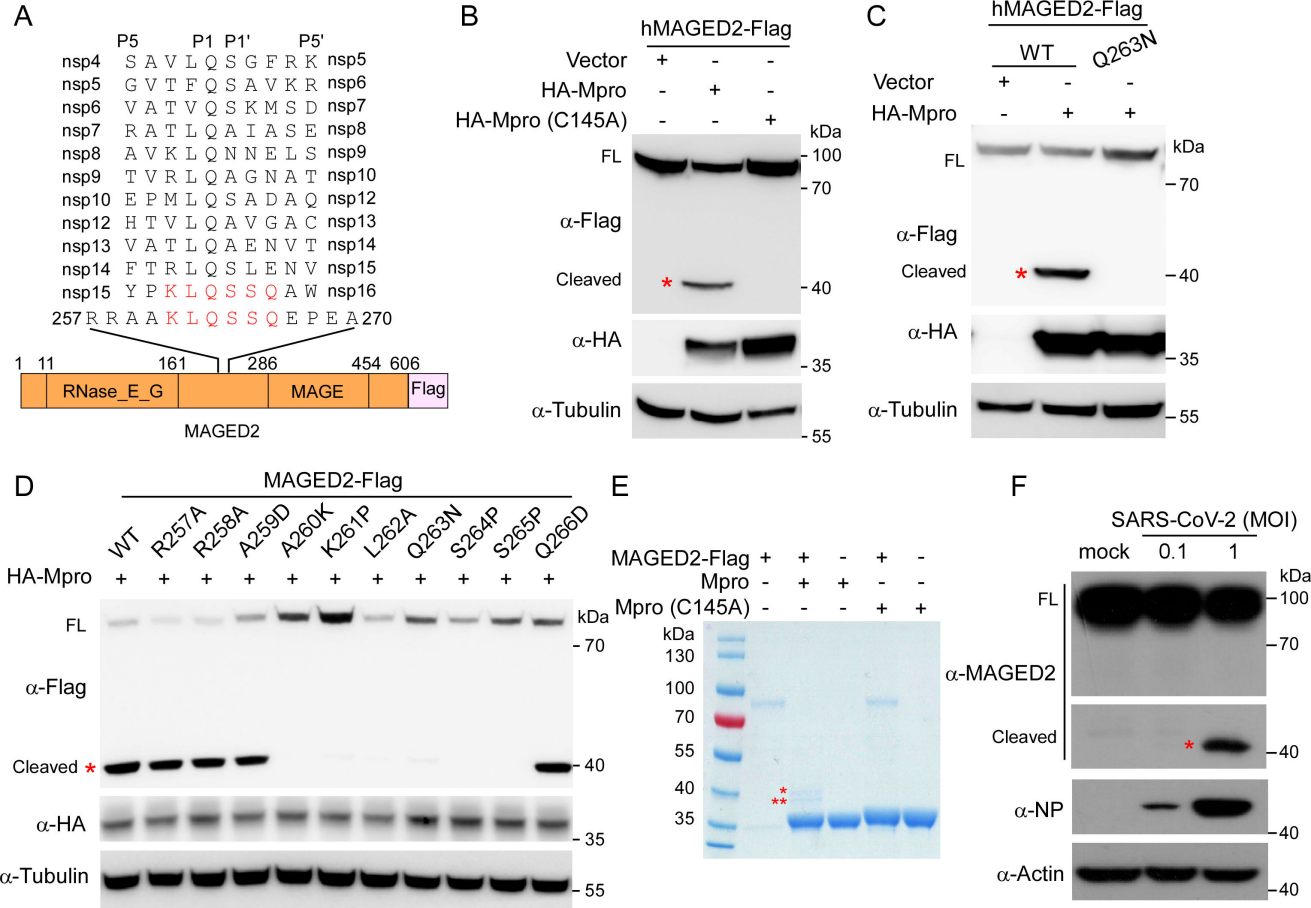
SARS-CoV-2 Mpro cleaves MAGED2 at Gln-263. (**A**) Putative Mpro cleavage sites in SARS-CoV-2 non-structural proteins and MAGED2. (**B and C**) HEK293T cells were co-transfected with human MAGED2 or Q263N mutant with Flag tag at C-terminal and HA-tagged SARS-CoV-2 Mpro or a proteolytically inactive mutant Mpro (C145A). Lysates from transfected cells were prepared for immunoblotting with antibodies, as indicated. (**D**) MAGED2 mutant with Flag tag at C-terminal and HA-tagged Mpro were co-expressed in HEK293T cells. Lysates from transfected cells were prepared for immunoblotting with antibodies, as indicated. (**E**) MAGED2 cleavage assay *in vitro*. Purified MAGED2 and Mpro wild-type (WT) or C145A mutant proteins were incubated *in vitro* and analyzed by Coomassie blue staining. One star is MAGED2_N_, and two stars indicate MAGED2_C_. (**F**) A549-hACE2 cells were infected with SARS-CoV-2 at a multiplicity of infection (MOI) of 0.1 or 1, and immunoblot was performed at 24-h post-infection. Red star indicates cleaved MAGED2. Each experiment was independently repeated three times with similar results, and the representative images are shown.

To identify the host proteins which could potentially be cleaved by SARS-CoV-2 Mpro, we analyzed the residues surrounding cleavage sites (P5-P5′) of Mpro within viral polyproteins (Fig. 1A; Fig. S1A). We therefore deduced that the sequence [VAEFGHRSTY]-[AVPT]-[TKRVM]-[LFV]-Q-[SAN]-[AGEIKLNSV] (P5-P2′) is the conserved cleavage motif, and Mpro catalyzes cleavage at Gln (P1)-(Ser/Ala/Asn) (P1′) peptide bonds, which may serve as a clue for identifying the putative targets of Mpro in a large-scale analysis. Next, we utilized GenomeNet motif search database (http://motif.genome.jp/) for profiling human proteins containing Mpro cleavage motif, and 353 host proteins were identified as putative targets of Mpro (Table S1).

Based on proteins’ function and their potential relevance in viral infection, we selected 12 proteins (MAGED2, SMARCA4, STAT4, STAT6, ACTN2, CDCA7, DNMT3B, NOP2, RETSAT, SLC25A22, TELO2, and STAT2) for cleavage validation. To this end, we constructed a series of cDNA of host proteins with a C-terminal Flag tag, and the cDNA of HA tagged SARS-CoV-2 Mpro. The recombinant constructs containing cDNA of each host gene and Mpro were transfected into HEK293T cells, respectively, and immunoblotting assay was performed to detect the cleavage of host proteins by Mpro. Among the 12 putative targets of Mpro, we only detected cleavage products in MAGED2 (Fig. S1B through D). Specifically, in the presence of Mpro, we observed the full-length 80 kDa MAGED2 and an additional band of about 40 kDa (Fig. S1B; Fig. 1B), which are consistent with a C-terminal region product of a putative cleavage occurring between 263 and 264 residues of MAGED2 (Fig. 1A and B). Moreover, the protease inactive mutant Mpro C145A is impaired in cleavage of MAGED2 (Fig. 1B, lane 3), suggesting that the cleavage is dependent on Mpro protease activity. To further test whether the cleavage site is at Gln-263 of MAGED2, we ectopically expressed Flag-tagged MAGED2 wild type (WT) or Q263N with HA-Mpro in HEK293T cells, respectively, to examine the cleavage of MAGED2 Q263N by Mpro. Immunoblotting assay result showed that Q263N mutant cannot be cleaved by Mpro (Fig. 1C, lane 3). To further establish the cleavage site of MAGED2 by Mpro, we overexpressed MAGED2-Flag and HA-Mpro in the HEK293T cells. After 3 days, we purified the cleaved product of MAGED2-Flag by Flag antibody bounds with magnetic beads and then analyzed the products using Edman degradation (Fig. S2A and 2B). Our results demonstrated that the first five residues at N-terminal of cleaved products of MAGED2 were S-S-Q-E-P (Fig. S2B), which were residues of 264th–268th of MAGED2. These results collectively demonstrated that Mpro could cleave MAGED2 at Q263. Furthermore, we made a series of MAGED2 mutants, which carried single substitution around the cleavage site of Q263 (P7-P3′), and the MAGED2 mutants exhibited varied sensitivities to Mpro cleavage (Fig. 1D). Substitutions within A260-S265 (P4-P2′) conferred resistance to Mpro cleavage (Fig. 1D), suggesting that these regions were key determinants of MAGED2 sensitivity to Mpro cleavage. To confirm that Mpro could directly cleave MAGED2, we purified recombinant WT Mpro, Mpro (C145A), and MAGED2, and incubated WT Mpro or Mpro (C145A) with MAGED2, respectively, for 2 h at 30°C. Subsequently, the mixtures were analyzed on SDS-PAGE. We observed two bands exclusively present in Mpro/MAGED2 mixture sample (Fig. 1E, lane 2), which was absent in the Mpro (C145A)/MAGED2 mixture sample (Fig. 1E, lane 4), and these results affirmed the direct cleavage on MAGED2 by Mpro. To validate the cleavage of MAGED2 by Mpro in the virus infection condition, we infected human lung carcinoma A549 cells expressing human ACE2 (A549-hACE2) with SARS-CoV-2 and the cleaved products of MAGED2 could be observed at a multiplicity of infection (MOI) of 1 (Fig. 1F). Taken together, these results demonstrated that SARS-CoV-2 Mpro is able to cleave human MAGED2 at Gln-263 residue.

### MAGED2 cleavage by Mpro is an evolutionarily conserved mechanism of coronavirus infection in mammals

**Fig 2:**
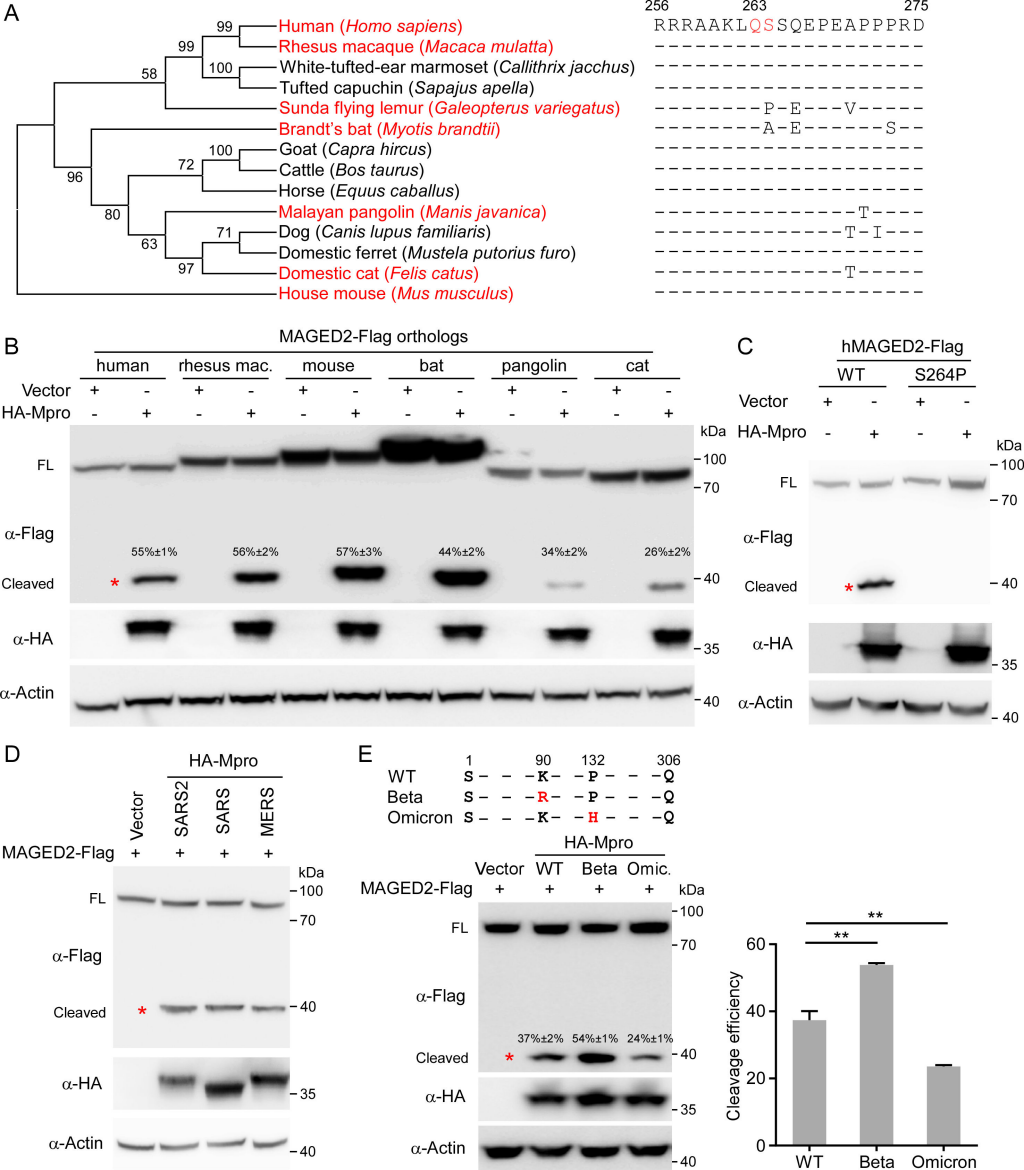
MAGED2 cleavage by Mpro is conserved in multiple mammalian species and coronaviruses. (**A**) A phylogenetic tree was constructed based on the protein sequences of MAGED2 orthologs by using the neighbor-joining method conducted in program MEGA6. MAGED2 residues neighboring Mpro cleavage site from human, rhesus macaque, White-tufted-ear marmoset, tufted capuchin, Sunda flying lemur, Brandt’s bat, goat, cattle, horse, Malayan pangolin, dog, domestic ferret, domestic cat, and house mouse are aligned. (**B to E**) HEK293T cells were transfected with MAGED2 orthologs as indicated species. The uncleaved or cleaved protein band intensity was quantitatively analyzed using ImageJ. Cleavage efficiency = cleaved products/(cleaved products + uncleaved protein) × 100% (**B**), MAGED2 mutant S264P (**C**), and HA-tagged Mpro from SARS-CoV-2, other coronavirus (SARS-CoV or MERS-CoV) (**D**), or SARS-CoV-2 variants (Beta or Omicron) (**E**). Lysates of transfected cells were analyzed by immunoblotting with the antibodies indicated on the left. Western blots are quantified with ImageJ. Each experiment was independently repeated three times with similar results, and the representative images are shown. Values are means plus standard deviations (error bars) from one representative experiment with three biological replicate samples. **, *P* < 0.01 by one-way analysis of variance.

To assess whether the Mpro-mediated MAGED2 cleavage has been evolutionarily conserved, we performed phylogenetic and evolutionary analyses of MAGED2 proteins. We found that MAGED2 is highly conserved across the 14 mammalian species. Especially, the residues around the cleavage site are highly conserved in these species (Fig. 2A), and residue Ala-264 in Brandt’s bat (*Myotis brandtii*) is different from others but still compliant with Mpro cleavage motif (Fig. 1A). However, ortholog from the Sunda flying lemur (*Galeopterus variegatus*) with proline (P) at its residue 264 (Fig. 2A) seems not compliant with Mpro cleavage motif (Fig. 1A). To investigate whether SARS-CoV-2 Mpro is able to cleave MAGED2 orthologs, we thus selected some representative species including rhesus macaque (*Macaca mulatta*), domestic cat (*Felis catus*), Brandt’s bat (*Myotis brandtii*), Malayan pangolin (*Manis javanica*), and house mouse (*Mus musculus*). Our results suggested that MAGED2 orthologs of all these tested species can be cleaved by SARS-CoV-2 Mpro, albeit with varying cleavage efficiency (Fig. 2B). Of note, the human MAGED2 S264P mutant, in which the 264th residue of hMAGED2 (S) was replaced with the corresponding Sunda flying lemur residue (P), was resistant to Mpro cleavage (Fig. 2C). Taken together, all these results suggest that the cleavage of MAGED2 by SARS-CoV-2 Mpro is highly conserved in multiple mammalian species.

Next, we sought to investigate whether cleavage of MAGED2 is conserved in other coronaviruses. For this purpose, we transfected HA-Mpro of SARS-CoV or MERS-CoV, together with human MAGED2 (MAGED2-Flag) into HEK293T cells. The immunoblotting results showed that SARS-CoV and MERS-CoV Mpro can cleave MAGED2 with comparable efficiencies of SARS-CoV-2 Mpro (Fig. 2D). The pandemic of COVID-19 continues, and new variants of SARS-CoV-2 persist to emerge (21, 22), and Beta and Omicron variants bear mutations in the Mpro (Fig. 2E, up panel). To compare the efficiencies of the cleavage of MAGED2 by Mpro from different variants, we co-expressed MAGED2 with Mpro from the original Wuhan strain (WT), Beta, or Omicron variants. Intriguingly, Mpro of Beta variant (K90R) cleaves MAGED2 more efficiently than the WT. In contrast, Mpro of Omicron (P132H) cleaves MAGED2 with less efficiency than WT (Fig. 2E). To glean more information about the varied cleavage efficiencies of Mpro to MAGED2, we turned to molecular dynamic stimulation to calculate the binding free energies of Mpro/MAGED2 complex which was predicted by AlphaFold-Multimer (23, 24) (Fig. S3A). Our analysis showed that the delta G binding of Mpro with MAGED2 is −87.31 ± 0.38 kcal/mol for WT, −104.23 ± 0.31 kcal/mol for Beta, and −73.08 ± 0.35 kcal/mol for Omicron (Fig. S3B and 3C), indicating that Mpro of Beta has the highest binding affinity with MAGED2, and Mpro of Omicron has the least binding affinity, which correlated with the varied cleavage efficiencies of various Mpro to MAGED2 (Fig. 2E). Collectively, MAGED2 cleavage by Mpro is an evolutionarily conserved mechanism of coronavirus infection in mammals.

### MAGED2 is a restriction factor that inhibits SARS-CoV-2 infection

**Fig 3:**
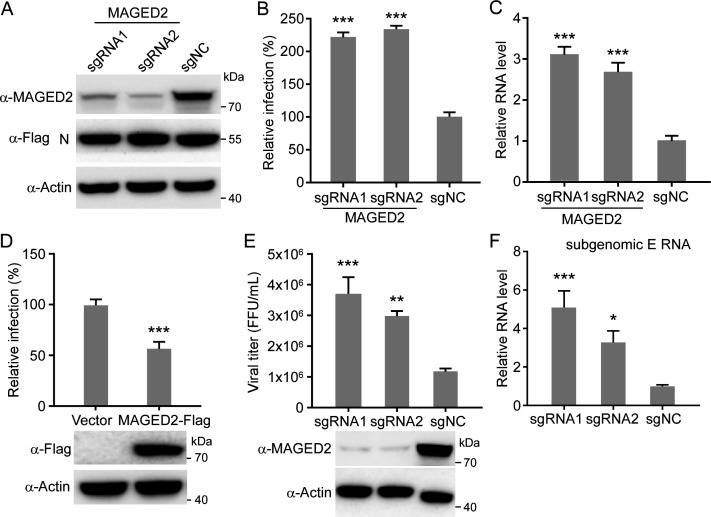
MAGED2 is a restriction factor that inhibits SARS-CoV-2 infection. (**A**) Caco-2-N cells were transduced with sgRNA targeting MAGED2. Whole-cell lysate was analyzed by immunoblotting assay at 5-day post-transduction. (**B and C**) WT or MAGED2-depleted Caco-2-N cells were infected with SARS-CoV-2 GFP/ΔN trVLP at an MOI of 0.1. After 24 h, cells were analyzed by flow cytometry to determine the percentage of SARS-CoV-2 GFP/ΔN trVLP-infected cells. Data are normalized with non-targeting control (**B**). Meanwhile, intracellular RNAs were purified for RT-qPCR assay to quantify SARS-CoV-2 genomic RNAs (**C**). (**D**) Human MAGED2 was ectopically expressed in Caco-2-N cells by lentiviral transduction, and the cells were subsequently infected with SARS-CoV-2 GFP/ΔN trVLP at an MOI of 0.1. Cells were analyzed by flow cytometry at 24-h post-infection to determine the percentage of the trVLP-infected cells. (**E and F**) MAGED2 knockout Caco-2 cells were infected with SARS-CoV-2 authentic virus at an MOI of 0.1. After 24 h, viral particles in the supernatant were titrated (**E**), and intracellular RNAs were purified for RT-qPCR assay to quantify SARS-CoV-2 subgenomic E RNAs (**F**). Values are means + standard deviations (error bars) from one representative experiment with three biological replicate samples, and each experiment was repeated three times. **, *P* < 0.01; ***, *P* < 0.001 by one-way analysis of variance.

MAGED2 is known to inhibit TRAIL-induced apoptosis by reducing cell surface expression of both receptors TRAIL-R1 and TRAIL-R2 (19, 25). However, MAGED2 function in virus infection is poorly understood. We utilized SARS-CoV-2 transcription and replication-competent virus-like particles (trVLP) system, which could recapitulate complete viral life cycle in human colon carcinoma Caco-2 cells expressing SARS-CoV-2 nucleocapsid protein (Caco-2-N) (26, 27) , to dissect the potential role of MAGED2 in SARS-CoV-2 infection via knockout (loss-of-function) and ectopic expression (gain-of-function) strategies. To this end, we knocked out MAGED2 by CRISPR/Cas9, and MAGED2 depletion did not affect viral N protein expression in Caco-2-N cells (Fig. 3A). Next, we infected WT or MAGED2 knockout Caco-2-N cells with SARS-CoV-2 GFP/ΔN trVLP (MOI = 0.1). After 24 h, cells were collected for flow cytometry or RT-qPCR analysis to quantify viral protein and RNA levels. Our results showed that MAGED2 knockout resulted in twofold increase of SARS-CoV-2 GFP/ΔN trVLP infection (percentage of GFP positive cells) compared with WT (non-targeting control) cells (Fig. 3B) and increased copies of viral genomic RNA and subgenomic RNA (Fig. 3C; Fig. S4A). Then, we ectopically expressed MAGED2 in the Caco-2-N cells (Fig. 3D), which were then infected with SARS-CoV-2 GFP/ΔN trVLP (MOI = 0.1). After 24 h, cells were collected for analysis of GFP expression. Our results showed that overexpression of MAGED2 inhibited SARS-CoV-2 infection, as evidenced by a decreased percentage of viral infection cells (Fig. 3D).

Next, we utilized the authentic SARS-CoV-2 virus (nCoV-SH01 strain) to infect the Caco-2 cells (MOI = 0.1) in which MAGED2 was knocked out by CRISPR/Cas9 or not. After 24 h of infection, the cell culture medium was collected for viral titer determination to quantify the production of progeny virus (Fig. 3E), and total cellular RNA was purified for RT-qPCR assay to specifically quantify the subgenomic E RNA (Fig. 3F), which is a biomarker to monitor actively replicating virus (28). Our result showed that MAGED2 depletion led to increase of progeny virus titer by three- to fourfolds (Fig. 3E) and subgenomic E RNA copy number by three- to fivefolds (Fig. 3F) accordingly.

Collectively, these results suggest that MAGED2 could restrict SARS-CoV-2 infection.

### MAGED2 inhibits SARS-CoV-2 genome replication but not restricts viral entry, assembly, and release

**Fig 4:**
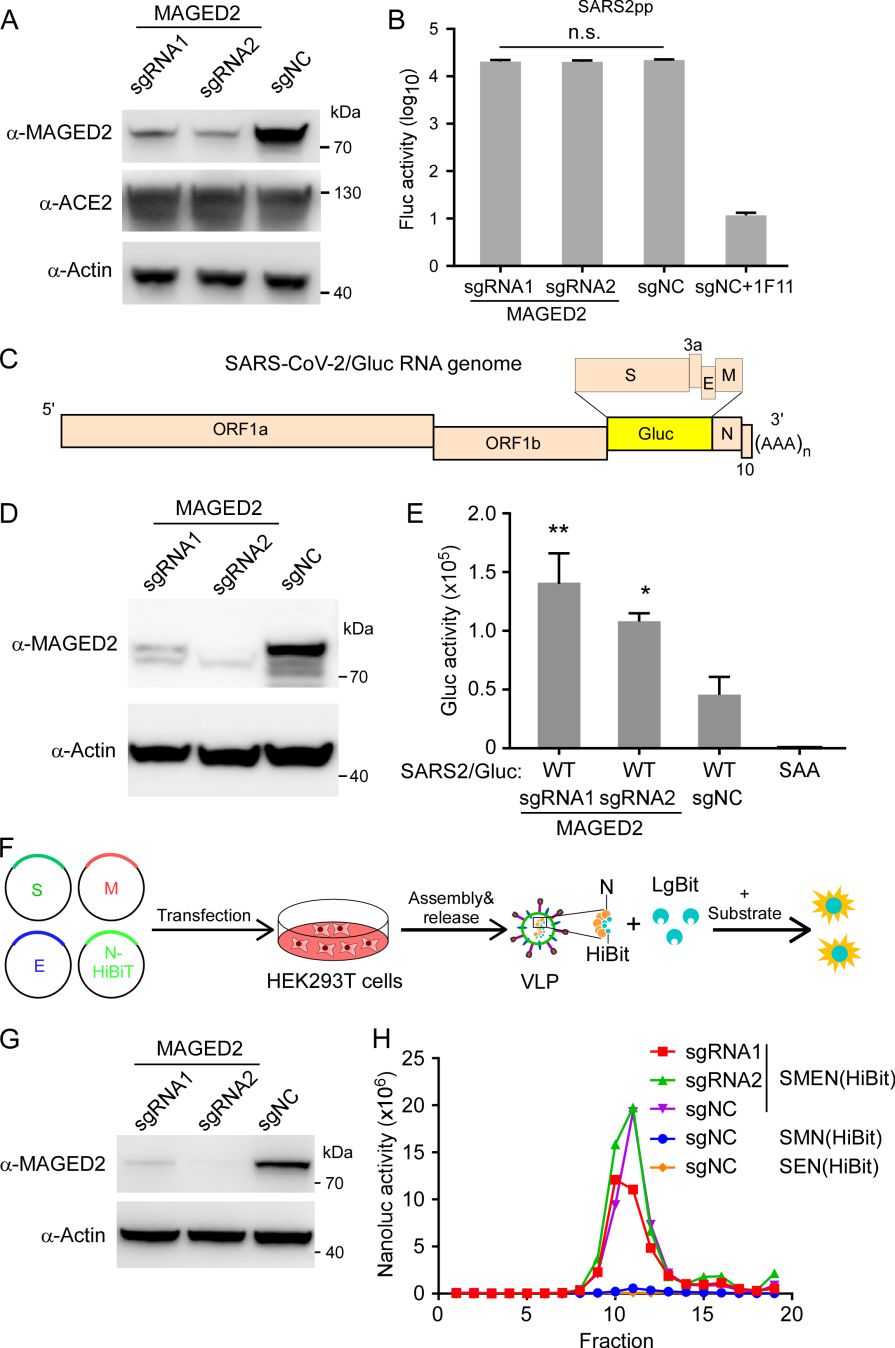
MAGED2 inhibits SARS-CoV-2 genome replication but not restricts viral entry, assembly, and release. (**A and B**) HeLa-ACE2 cells were transduced with sgRNA lentivirus targeting MAGED2. Whole-cell lysates were analyzed by immunoblot with MAGED2, ACE2, and β-actin antibodies. The HeLa-ACE2 cells with or without MAGED2 depletion were infected with MLV retroviral particles (Fluc as the reporter) pseduotyped with SARS-CoV-2 spike (MLV SARS-CoV-2pp). Fluc activity was measured at 48-h post-infection. 1F11 is SARS-CoV-2 neutralizing antibody as the positive control. (**C**) Schematic representation of SARS-CoV-2 Gluc replicon RNA genome. (**D and E**) Caco-2-N cells were transduced with sgRNA lentivirus targeting MAGED2. Whole-cell lysates were analyzed by immunoblot with MAGED2 and β-Actin antibodies. Then, the cells with or without MAGED2 genetic ablation were transfected with SARS-CoV-2 Gluc WT or SAA (RdRp inactive mutant) replicon RNAs, and Gluc activity was assayed at 48-h post-transfection. (**F**) Schematic representation of VLP production and detection. (**G and H**) HEK293T cells were transduced with sgRNA lentivirus targeting MAGED2. Whole-cell lysates were analyzed by immunoblot with MAGED2 and β-actin antibodies. Then, the HEK293T cells with or without MAGED2 knockout in 10 cm dish were transfected with equal amounts of plasmids (24 µg in total) encoding the SARS-CoV-2 S, E, M, and HiBiT-N proteins. After 24 h, cell culture supernatants were collected. VLPs separated by 10%–60% sucrose gradient centrifugation were measured with Nano-Glo luciferase kit. All data are representative of three independent experiments. Values are means + standard deviations (error bars) (*n* = 3). *, *P* < 0.05; **, *P* < 0.01; n.s., not significantly different by one-way analysis of variance.

The above findings suggest that MAGED2 could inhibit SARS-CoV-2 infection, we therefore sought to dissect which step of viral life cycle is blocked by MAGED2. We initially investigated whether MAGED2 affected SARS-CoV-2 entry by examination of cell entry of virion pseudotyped with SARS-CoV-2 spike proteins. To this end, we generated murine leukemia virus (MLV) retroviral particles (Fluc as the reporter) pseudotyped with SARS-CoV-2 spike (MLV SARS-CoV-2pp) (29, 30) to infect WT or MAGED2 knockout HeLa-ACE2 cells (Fig. 4A). Our results suggested that the depletion of MAGED2 did not affect SARS-CoV-2 cell entry (Fig. 4B).

Next, we utilized SARS-CoV-2 replicon and VLP models to examine the role of MAGED2 in viral genome replication and assembly/release steps, respectively. SARS-CoV-2 Gluc replicon RNA, in which viral structure genes S, E, and M were replaced with a secretory Gaussia luciferase (Gluc) gene (Fig. 4C), was transfected into Caco-2-N cells knockout with MAGED2 or not (Fig. 4D). Cells were washed with phosphate-buffered saline (PBS) at 24-h post-transfection, and Gluc activity was measured at 48-h post-transfection. MAGED2 knockout led to two- or threefold increases of luciferase activity compared with control cells (Fig. 4E), suggesting that MAGED2 inhibits SARS-CoV-2 genomic RNA replication. SARS-CoV-2 VLP can be produced by co-expression of structure proteins S, E, M, and N, and this system can be used to evaluate SARS-CoV-2 assembly and release (31 - 34). To make the production of VLP measurable, we constructed N tagged with HiBiT according to the previous study (35). Briefly, the recombinant HiBiT-tagged nucleocapsid protein is co-expressed with other SARS-CoV-2 structural proteins (S, E, and M), and secreted HiBiT levels reflect VLP assembly and secretion (Fig. 4F). Therefore, the plasmids encoding S, E, M, or HiBiT-tagged N were co-transfected into WT or MAGED2-depleted HEK293T cells (Fig. 4F and G), and VLP production was measured at 24-h post-transfection. VLP in different fractions after density gradient centrifugation was evaluated by measuring Nanoluc luciferase activity (Fig. 4H). MAGED2 knockout did not increase VLP production (especially fraction 11) (Fig. 4H), suggesting that MAGED2 did not interfere with SARS-CoV-2 assembly and release. Collectively, these results demonstrated that MAGED2 restricts SARS-CoV-2 infection via inhibition of SARS-CoV-2 genomic RNA replication but not viral entry, assembly, or release.

### MAGED2 interacts with viral N protein to disturb the association of N with viral genome

To dissect the detailed mechanism of MAGED2 restricting SARS-CoV-2 replication, we examined the association of MAGED2 with SARS-CoV-2 proteins, which are relevant to viral genome replication (non-structural proteins and viral N) by co-immunoprecipitation (co-IP) assay. We found that MAGED2 was associated with SARS-CoV-2 nsp9, nsp12, and N protein (Fig. S5A through C). Nsp12 is the catalytic subunit of viral RNA-dependent RNA polymerase (RdRp) complex which executes transcription and replication of the viral RNA (36). Two cofactor subunits, nsp7 and nsp8, are associated with nsp12 to constitute an obligatory core polymerase complex to confer processivity for RNA (37). We therefore tested whether MAGED2 can regulate the assembly of polymerase complex by co-IP assay. Our data demonstrated that MAGED2 did not affect the interaction among nsp7, nsp8, and nsp12 (Fig. S6A). Consistently, the presence of recombinant MAGED2 did not affect *in vitro* polymerase activity of nsp12-nsp7-nsp8 complex (Fig. S6B and 6C).

**Fig 5:**
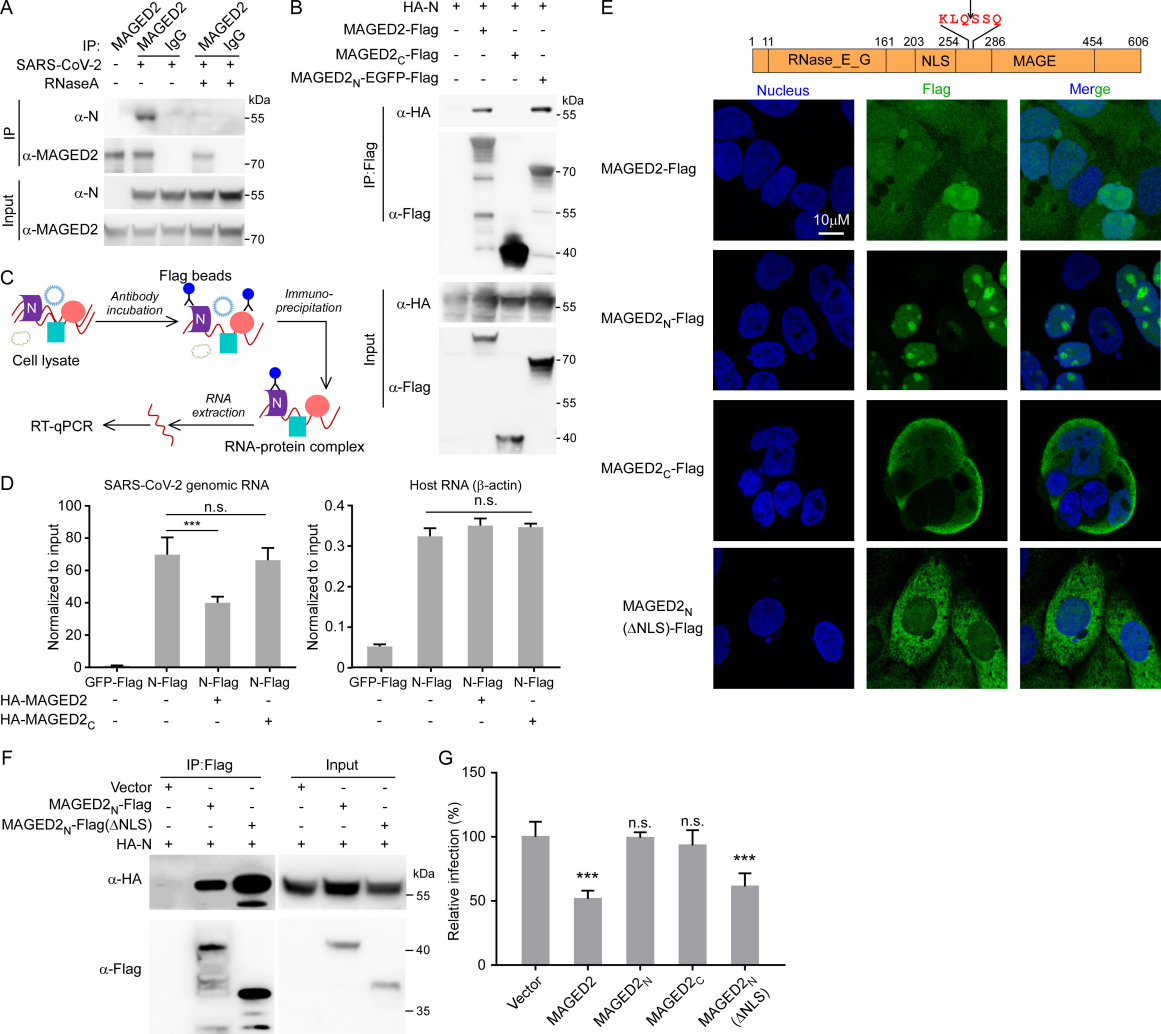
MAGED2 interacts with viral N protein to disturb the association of N with viral RNA genome. (**A**) Caco-2 cells were infected with SARS-CoV-2 authentic virus at an MOI of 0.1. After 24 h, cells were collected and lysed. Cell lysates were immunoprecipitated with MAGED2 antibody or IgG control with/without 50 µg/mL RNase A treatment. Immunoprecipitants were subjected for immunoblotting assay with MAGED2 and N antibodies. (**B**) HA-N protein and Flag tagged MAGED2, MAGED2_N_ (1-263 aa)-EGFP or MAGED2_C_ (264-606 aa) were transfected into HEK293T cells. After 48 h, cell lysates were immunoprecipitated by Flag antibody-conjugated magnetic beads. Immunoprecipitants were subjected for immunoblotting assay with Flag and HA antibodies. (**C and D**) Schematic representation of RNA immunoprecipitation (RIP) assay. SARS-CoV-2 Gluc replicon RNAs, plasmids encoding GFP-Flag or N-Flag and HA-MAGED2 full-length or C-terminal truncation were co-electroporated into HEK293T cells. RIP was performed at 24-h post-electroporation as indicated, and RT-qPCR assay was conducted to determine the RNA abundances. The precipitated RNA was normalized with input. (**E**) Subcellular localization of MAGED2 full-length and its truncations. Flag-tagged MAGED2 full-length, MAGED2_N_, MAGED2_C_, or MAGED2_N_(ΔNLS) was expressed in Caco-2 cells by lentiviral transduction. Cells were stained with Flag antibody, and the nuclei were stained with DAPI. (**F**) HA-tagged N protein and Flag-tagged MAGED2_N_ (1-263 aa) or MAGED2_N_ (ΔNLS) were transfected into HEK293T cells. After 48 h, cell lysates were immunoprecipitated by Flag antibody-conjugated magnetic beads. Immunoprecipitants were subjected for immunoblotting assay with Flag and HA antibodies. (**G**) Human MAGED2 full-length or its truncations were ectopically expressed in Caco-2-N cells by lentiviral transduction, and the cells were subsequently infected with SARS-CoV-2 GFP/ΔN trVLP at an MOI of 0.1. Cells were analyzed by flow cytometry at 24-h post-infection to determine the percentage of the trVLP-infected cells. Values are means + standard deviations (error bars) (*n* = 3). ***, *P* < 0.001; n.s., not significantly different by one-way analysis of variance.

N protein is required for coronavirus genome replication and subgenomic RNA transcription by association with viral RNA (38 - 41). We next characterized the association of viral N protein with MAGED2. Consistent with our previous results in this study (Fig. S5C; Fig. 5A), MAGED2 could associate with viral N protein, which was further evidenced by the co-localization of endogenous MAGED2 with viral N protein in Caco-2 cells (Fig. S7A). In addition, the interaction of endogenous MAGED2 with viral N protein could also be detected in the viral infection context, and it seems that the interaction is dependent on RNA as the interaction could be disrupted by RNase A treatment (Fig. 5A). Since the MAGED2 was cleaved by Mpro at Gln-263, we thus dissected the interaction of the cleaved products of MAGED2 with N protein. We transfected full-length MAGED2-Flag, MAGED2_N_-EGFP-Flag (MAGED2 N-terminal products [1–263 aa] fused with EGFP-Flag); due to unknown reason, the MAGED2_N_-Flag was not stable (Fig. S7B), and EGFP fusion could stabilize MAGED2_N_ (Fig. 5B, lane 4), or MAGED2_C_-Flag (MAGED2 C-terminal products [264–606 aa] fused with Flag) with HA-N into HEK293T cells, and then performed co-IP with anti-Flag antibody to detect the interaction of MAGED2 or its mutants with HA-N (Fig. 5B). Our results showed that MAGED2_N_ is critical and sufficient to mediate MAGED2 association with viral N protein (Fig. 5B; Fig. S7B).

**Fig 6:**
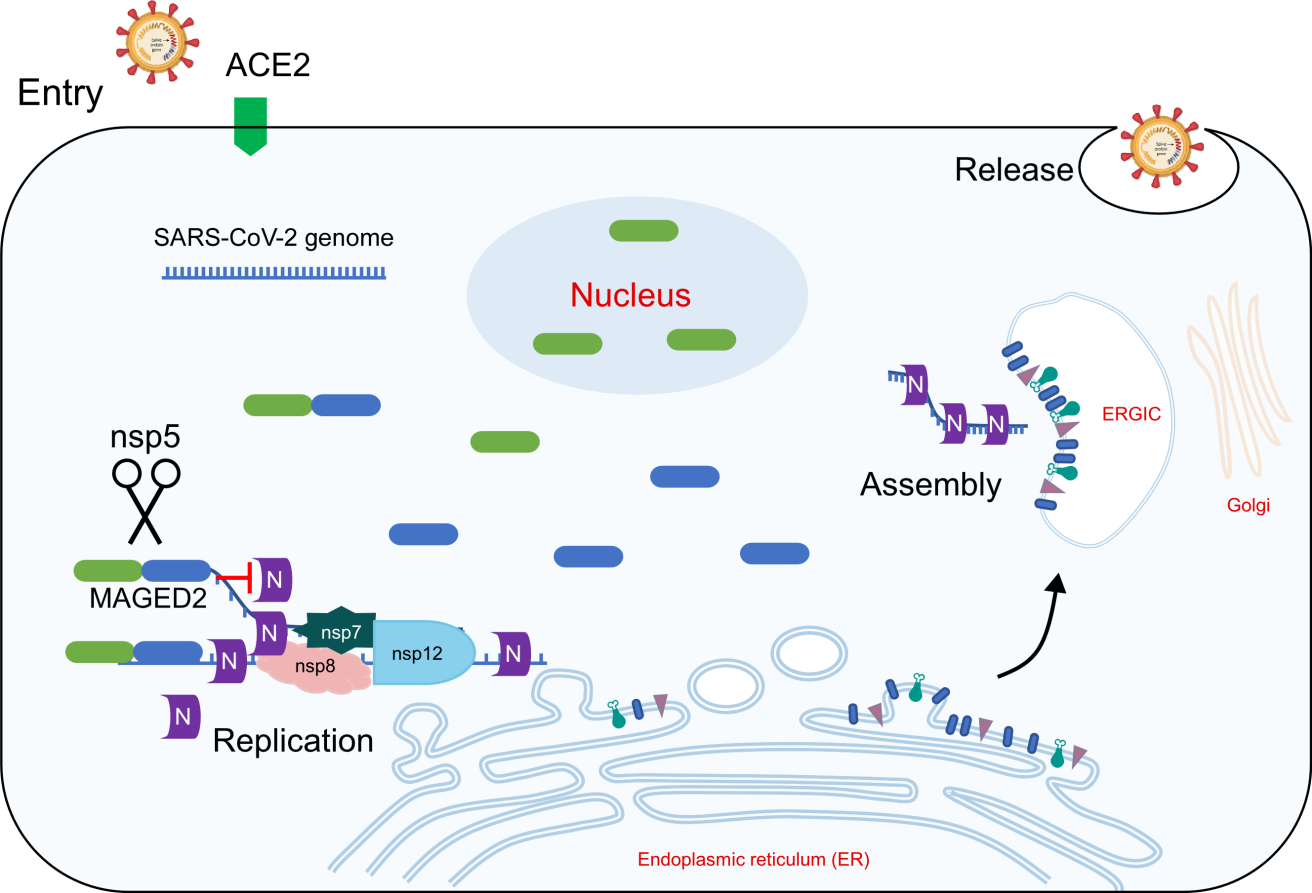
Mpro cleaves MAGED2 to antagonize its antiviral activity. Model depicts that MAGED2 restricts SARS-CoV-2 replication by decreasing the interaction between N protein and viral genome through its N-terminal region. Mpro cleaves MAGED2 at Gln-263, and MAGED2_N_ translocated into the nucleus, which relieving its antiviral effect.

Because RNA mediates the association between MAGED2 and nucleocapsid protein (Fig. 5A), we thus hypothesize that MAGED2 binds viral genomic RNA and disturbs the association of N protein with viral genome, thereby inhibiting viral replication. To test this hypothesis, RNA immunoprecipitation (RIP) assay was performed to detect the interaction between SARS-CoV-2 genomic RNA and N protein in the presence or absence of MAGED2 (Fig. 5C; Fig. S7C) . RIP-qPCR results showed that N-Flag exhibited specific interaction with viral genomic RNA (approximate 60-fold enrichment of viral RNA over input) (Fig. 5D, left panel), whereas N-Flag exhibited negligible interaction with host mRNA such as β-actin mRNA (approximate 0.3- to 0.4-fold enrichment over input) (Fig. 5D, right panel). Furthermore, our results also demonstrated that overexpression of MAGED2 could severely impaired the interaction between N and SARS-CoV-2 genomic RNA (Fig. 5D, left panel). However, the MAGED2_C_-Flag, which is not able to interact with viral N protein (Fig. 5B), exhibited negligible inhibition in the interaction between N and viral genomic RNA (Fig. 5D, left panel). It has been reported that MAGED2 localizes in both nucleus and cytoplasm, and MAGED2 (1-263 aa) contains nuclear localization signal sequences (NLSs) (17). We hypothesized that upon cleavage of MAGED2 by Mpro, the MAGED2_N_ translocates into the nucleus, compromising its interference with viral N protein. To test this, Flag-tagged MAGED2, MAGED2_N_, MAGED2_C_, or MAGED2_N_ without NLS by deletion of 176th–248th residues (MAGED2_N_(ΔNLS)-Flag) was expressed in Caco-2 cells, respectively, and immunofluorescence assay was performed to observe the localization of MAGED2 mutants. Consistent with the previous study (17), MAGED2 localized in both nucleus and cytoplasm (Fig. 5E). However, MAGED2_N_ was exclusively localized in the nucleus; in contrast, MAGED2_C_ and MAGED2_N_(ΔNLS)-Flag were distributed in cytoplasm (Fig. 5E). We next tested the antiviral effects of MAGED2, MAGED2_N_, MAGED2_C_, or MAGED2_N_(ΔNLS) using SARS-CoV-2 trVLP cell culture model (26, 27). The Caco-2-N cells were transduced with lentiviruses expressing MAGED2-Flag, MAGED2_N_-Flag, MAGED2_C_-Flag, or MAGED2_N_(ΔNLS)-Flag. SARS-CoV-2 GFP/ΔN trVLP infected the Caco-2-N cells expressing MAGED2-Flag or mutants (MOI = 0.1), and then GFP was analyzed after 24 h of infection (Fig. 5F and G). Our results showed that MAGED2_N_-Flag, which localized in nucleus (Fig. 5E), and MAGED2_C_-Flag, which localized in cytoplasm (Fig. 5E) but was not able to interact with viral N protein (Fig. 5B), exhibited negligible antiviral activity. However, MAGED2_N_(ΔNLS)-Flag, which localized in cytoplasm and was able to interact with viral N protein (Fig. 5E and F), exhibited comparable antiviral activity against SARS-CoV-2 trVLP infection with that of WT MAGED2 (Fig. 5G). These results suggested that Mpro cleaves MAGED2 to reprogram its subcellular localization to relieve the inhibition on viral replication (Fig. 6).

## DISCUSSION

Potential host range of SARS-CoV-2 has been reported to be broad from both structural and functional analyses of ACE2 (42 - 44). SARS-CoV-2 infection needs to overcome host antiviral response. Interferon signaling is the first line of host to defend viral infection by inducing the expression of hundreds of interferon-stimulated genes, which exert antiviral activity in various stages of viral life cycle (45, 46). Multiple SARS-CoV-2 proteins have been reported to inhibit type I interferon signaling pathway, including nsp1, nsp3, Mpro, nucleocapsid protein, ORF3a, ORF7, and ORF9b (47). Mpro, which is one of the most conserved proteins between SARS-CoV and SARS-CoV-2 responsible for viral polypeptide possessing, is an appealing target to develop antivirals for SARS-CoV-2 treatment (48). Nirmatrelvir, the inhibitor of Mpro developed by Pfizer, has been approved by FDA to treat mild-to-moderate COVID-19 patients (49). In this study, we found that MAGED2 is a restriction factor of SARS-CoV-2 replication by decreasing the interaction of nucleocapsid with viral genome, and Mpro is able to cleave MAGED2 at its Gln-263. After the cleavage, the cleaved product of MAGED2 translocates into nucleus, which relieves the restriction on viral N protein in the cytoplasm (Fig. 6). Our findings reveal a novel mechanism for SARS-CoV-2 antagonizing host antiviral response by reprogramming of subcellular localization of host proteins.

SARS-CoV-2 Mpro has been reported to cleave multiple host factors, including TAB1, NLRP12, RIG-I, NEMO, and RNF20, to inhibit host antiviral response (12 - 15). It would be of great significance to build a database (cleave-omics), including the host proteins, which could be cleaved by SARS-CoV-2 Mpro for comprehensive understanding of the virus-host interaction. Previous studies and our study here have identified multiple host proteins in the antiviral response that could be cleaved by viral Mpro, implying that viral protease-mediated host protein cleavage is another critical mechanism utilized by virus for immune evasion. In addition, it is conceivable that the host proteins participating in important physiological processes, such as growth, development, metabolism, and reproduction could also be cleaved by viral protease, which subsequently disturb the normal physiological process, eventually leading to disease progression. Thus, viral protease-mediated host protein cleavage could be an important determinant of viral pathogenicity (50). It is critical to develop a high-throughput proteomic approach to profile the host proteins cleaved by the viral protease, which will provide more insights into viral immune evasion and pathogenicity.

MAGED2 is highly conserved in different mammalian species (51). Mutations in MAGED2 cause transient Bartter syndrome characterized by perinatal loss of urinary concentration capability and large urine volumes (20). MAGED2 is expressed universally in normal tissues and participates in cell cycle regulation (52). In addition, MAGED2 is able to suppress the expression of TRAIL death receptor 2 (TRAIL-R2) and plays an important role in protecting melanoma cells from apoptosis induced by TRAIL (19), thus it has been recognized as a cancer diagnostic marker (52). However, functional information about MAGED2 is poorly understood. In this study, we demonstrated that MAGED2 was a novel antiviral factor against SARS-CoV-2. In addition, our present study provided evidence that Mpro from different mammalian CoVs cleaves MAGED2 at Gln-263 and that the cleaved products translocate into nuclei, thus losing the ability to inhibit viral genomic RNA replication in the cytoplasm. Thus, cleaving MAGED2 may be a common strategy used by different mammalian CoVs to antagonize the antiviral role of MAGED2. In contrast, the NS2B3 protease of flaviviruses, such as ZIKV, DENV, WNV, and YFV, could cleave STING in a species-dependent manner (53). The cleavage site of human STING by NS2B3 protease is not conserved, and mouse STING is resistant to NS2B3 cleavage, which could potentially be the genetic determinant of ZIKV, as well as other flavivirus host range (53). However, MAGED2 cleavage by Mpro is an evolutionarily conserved mechanism of coronavirus infection in mammals; therefore, inhibition of the cleavage of MAGED2 by Mpro represents a novel target for the development of broad-spectrum anti-CoVs drugs, and this is worthy of further study.

In addition, further studies were required for full understanding of antiviral effects of MAGED2. As presented in this study, we only examine the antiviral effect of MAGED2 on SARS-CoV-2, and it will be of great interest to test its antiviral activity against other coronavirus, as well other RNA viruses. As we found here, MAGED2 constitutively expressed, which is different from IFN-stimulating genes that are induced by IFN stimulation, and acts as an intrinsic restriction factor of SARS-CoV-2 infection. Thus, the mechanisms governing MAGED2 transcription were worthy of future investigation. Furthermore, given the cleavage efficiencies of MAGED2 by SARS-CoV-2 and variants were varying, the relationship of MAGES2 cleavage with viral transmission or pathogenicity was needed to be established.

It is important to point out that our study has limitations. First, the restrictive effect of MAGED2 on SARS-CoV-2 infection is not very potent. As shown in Fig. 3, depletion of endogenous MAGED2 results in two- to threefold increase of SARS-CoV-2 trVLP or three- to fourfold of authentic SARS-CoV-2 replication *in vitro*. It will be worthy to demonstrate its antiviral activity *in vivo* in the future. Second, the biological consequences of the interaction between MAGED2 and viral RdRp nsp12 remain elusive (Fig. S5A and B). Our results suggest that the interaction has negligible effect on holo-RdRp complex assembly and *in vitro* polymerase activity (Fig. S6A through C); however, whether the interaction could alter holo-RdRp complex activity *in vivo* required to be further explored.

Collectively, we described the novel restriction factor MAGED2 and showed the evolutionarily conserved interaction between coronavirus Mpro and MAGED2, which contributes to understanding viral immune evasion mechanisms and developing novel antiviral drugs.

## MATERIALS AND METHODS

### Cell culture and SARS-CoV-2 virus

HEK293T, HeLa, and Caco-2 cells were maintained in Dulbecco’s modified Eagle medium (Gibco, China) supplemented with 10% (vol/vol) fetal bovine serum and 50 IU/mL penicillin/streptomycin in a humidified 5% (vol/vol) CO_2_ and 37°C incubator. All cells in this study were tested negative for mycoplasma. The SARS-CoV-2 strain nCoV-SH01 (GenBank accession no. MT121215, https://www.ncbi.nlm.nih.gov/nuccore/MT121215) was isolated from a COVID-19 patient and propagated in Vero E6 cells for use. All experiments involving virus infections were performed in the biosafety level 3 facility of Fudan University following all regulations.

### Plasmid construction and transfection

To construct sgRNA targeting MAGED2, the plentiCRISPRv2 empty vector was digested with BsaI-HF version2 restriction enzyme. Plasmid backbone was extracted with gel extraction kit (Omega), and annealed sgRNA fragments were inserted into backbone by T4 DNA ligase (NEB). sgRNA target sequences are listed in Table S2. Host protein (MAGED2, SMARCA4, STAT4, STAT6, ACTN2, CDCA7, DNMT3B, NOP2, RETSAT, SLC25A22, TELO2, and STAT2) expressing plasmids were purchased from WZ Biosciences (China). SARS-CoV-2 proteins, MAGED2 full-length, and its truncation expressing plasmids were constructed into pLVX-IRES-zsGreen1 by 2× MultiF Seamless Assembly Mix (RK21020, Abclonal, China). All plasmids were confirmed by Sanger sequencing. Plasmids were transfected into HEK293T cells by VigoFect DNA transfection reagent (Vigorous Biotechnology) in 150 mM NaCl following standard protocol.

### Lentivirus production and transduction

Vesicular stomatitis virus G protein pseudotyped lentiviruses were produced by co-transfection of pMD2.G (12259; Addgene), psPAX2 (12260; Addgene), and the transfer vector in HEK293T cells. Transfection was using Vigofect (Vigorous Biotechnology) following standard protocol. Culture medium was changed 12-h post-transfection, and supernatants containing lentivirus were collected at 36-, 60-, and 84-h post-transfection. Cell debris was cleared by centrifugation at 4,000 rpm for 10 min. Supernatants were aliquoted and frozen in −80°C refrigerator. For transduction, the cells were infected with lentivirus in the presence of 10 µg/mL polybrene. Supernatants were changed 12-h post-infection.

### Co-immunoprecipitation and western blotting

Proteins tagged with HA and Flag separately were co-transfected into HEK293T cells. Forty-eight hours post-transfection, the cells were collected by trypsin digestion and washed with PBS two times. The cells were lysed by cell lysis buffer (50 mM Tris-HCl [pH 7.5], 150 mM NaCl, 1 mM EDTA, and 1% NP-40) supplied with 1 mM dithiothreitol (DTT), 1 mM phenylmethylsulfonyl fluoride (PMSF), and protease inhibitor. Cells were lysed for 30 min at 4°C with rotation, and the lysates were cleared at 13,500 rpm and 4°C for 15 min. About 50 µL whole-well lysate was collected for western blot analysis. The remaining supernatants were incubated with Flag M2 magnetic beads (Sigma-Aldrich, M8823) at 4°C overnight. Beads were washed with cell lysis buffer for five times, then eluted with 60 µL 1× sodium dodecyl sulfate (SDS) loading buffer by heating for 10 min at 95°C. SDS-polyacrylamide gel electrophoresis (SDS-PAGE) immunoblotting was performed as follows: protein samples were electrophoresed in 4%–12% polyacrylamide gels and transferred onto polyvinylidene difluoride (PVDF) membrane. The membranes were blocked with 5% non-fat milk in 1× tris-buffered saline (TBS) containing 0.1% (vol/vol) Tween 20 at room temperature for 30 min. The membranes were exposed to primary antibodies: anti-MAGED2 (15252-1-AP, Proteintech), β-tubulin (CW0098, CWBIO), Flag (F1804 and F7425, Sigma-Aldrich), HA-HRP (M20021, Abmart), and β-actin (AM1021b, Abcepta) in 5% non-fat milk in 1× TBS containing 0.1% Tween 20 for 1.5 h. The blots were then washed in 1× TBS containing 0.1% Tween 20 three times. After 1 h exposure to HRP-conjugated secondary antibodies, subsequent washes were performed. Membranes were exposed using the Luminescent image analyzer (GE).

### RT-qPCR

RNAs were extracted with TRIzol Reagent (Invitrogen) following standard protocol (26). RNAs were dissolved in 50 µL nuclease-free water. qPCR primers of target genes are listed in Table S2. Briefly, 1 µg of total RNAs was reverse transcribed using ReverTra Ace qPCR RT Kit (FSQ-101, TOYOBO, Japan). qPCR reactions were carried out using the 2× RealStar Green Power Mixture (Genstar, A311) according to the instruction. The relative expressions of the target genes were calculated using the 2^−ΔΔCt^ method.

### *In vitro* transcription and RNA transfection

SARS-CoV-2 Gluc replicon templates were prepared by *in vitro* ligation according to the previous study (26). Then, it was transcribed with mMESSAGE mMACHINE T7 Transcription Kit (Thermo Fisher Scientific). Viral replicon RNA was transfected using TransIT-mRNA transfection reagent (Mirus Bio) according to the instructions.

### RIP assay

About 1 × 10^7^ HEK293T cells were electroporated with plasmids expressing GFP-Flag, N-Flag, HA-MAGED2, and SARS-CoV-2 Gluc replicon RNA. Twenty-four hours post-electroporation, the cells were lysed in ice-cold RIP lysis buffer (50 mM Tris-HCl [pH 7.5], 150 mM NaCl, 1% Triton X-100, 5% glycerol, supplemented with 1 mM DTT, 1 mM PMSF, 1:100 P.I. cocktail, and 1:100 RNase Inhibitor) for 0.5 h at 4°C with constant rotation. RQ1 DNase was supplied into the lysates to incubate at 37°C for 10 min for DNA digestion. The lysates were cleared by centrifugation at 13,000 rpm and 4°C for 15 min. About 7.5% of the cell lysate was saved as input for RT-qPCR. Cell lysates were incubated with Flag M2 magnetic beads at 4°C overnight. After 4 × 5 min washes by RIP200 buffer (20 mM Tris-HCl [pH 7.4], 200 mM NaCl, 1 mM EDTA, 0.3% Triton X-100, and 5% glycerol), RNAs were eluted in proteinase K digestion buffer [50 mM Tris-HCl (pH 7.4), 150 mM NaCl, 0.5% SDS, and 5 mM EDTA containing 20 µg proteinase K) at 55°C for 1 h (54). The RNA was extracted by TRIzol Reagent (Invitrogen) according to the manufacturer’s protocol. Reverse transcription was conducted using ReverTra Ace qPCR RT Kit (TOYOBO) with random primers. Quantitative real-time PCR was performed with 2× RealStar Green Power Mixture (Genstar) according to the instruction. Primers are listed in Table S2.

### SARS-CoV-2 GFP/ΔN trVLP infection

SARS-CoV-2 GFP/ΔN trVLPs were produced in Caco-2 cells expressing SARS-CoV-2 nucleocapsid protein (Caco-2-N) reported in the previous study (26). WT or MAGED2 knockout Caco-2-N cells were infected with SARS-CoV-2 GFP/ΔN trVLPs at an MOI of 0.1, SARS-CoV-2 GFP/ΔN trVLP infections were analyzed by flow cytometry or RT-qPCR at 24- or 48-h post-infection.

### SARS-CoV-2 authentic virus infection and titration

Caco-2 cells were seeded one night prior to infection by SARS-CoV-2 (nCoV-SH01) at an MOI of 0.1. Twenty-four hours post-infection, supernatants were collected and titrated in Caco-2 cells. For viral titration, Caco-2 cells were seeded into 96-well plate 1 day before and infected with diluted SARS-CoV-2. One day post-infection, the cells were fixed and stained with house-made mouse anti-SARS-CoV-2 nucleocapsid protein serum (1:1,000) at 4°C overnight. The number of N positive foci was counted and used to calculate infectious titer.

### VLP production and detection

WT or MAGED2 knockout HEK293T cells in 10 cm dish were co-transfected with equal amounts of plasmids (24 µg in total) encoding the SARS-CoV-2 S, E, M, and HiBiT nucleocapsids using Vigofect transfection reagent (T001, Vigorous Biotechnology, China) following standard protocol. Supernatant containing VLPs were collected at 24-h post-transfection, and cell debris was removed by centrifugation at 4,000 rpm, 4°C for 30 min. VLPs in supernatant were then concentrated by 20% sucrose centrifugation at 100,000 × *g*, 4°C for 3 h. Pellets were dissolved in PBS and then separated by 10%–60% sucrose gradient centrifugation at 100,000 × *g*, 4°C for 3 h. VLPs in different fractions were measured by Nano-Glo luciferase assay reagents (N1110, Promega, CA, USA). Briefly, aliquots of each fraction were mixed with LgBiT protein and Nano-Glo luciferase assay substrate (Promega). Nluc activity was measured by GloMax Discover System (Promega).

### Production of SARS-CoV-2 S pseudotyped virus and virus entry

Pseudovirions were produced by co-transfection HEK293T cells with retroviral vector pTG-MLV-Fluc, pTG-MLV-Gag-pol, and pcDNA3.1 expressing the SARS-CoV-2 spike using VigoFect (Vigorous Biotechnology) (29). The supernatants were harvested at 24- and 48-h post-transfection, centrifuged at 4,000 rpm for 5 min to remove cell debris and kept at −80°C. Virus entry was assessed by transduction of pseudoviruses in HeLa-hACE2 cells with/without MAGED2 knockout in 48-well plates. After 48 h, intracellular luciferase activity was determined using the Luciferase Assay System (Promega, Cat. #E1500) according to the manufacturer’s instructions. Luminescence was recorded on a GloMax Discover System (Promega). All experiments were performed in triplicates.

### Protein expression and purification

Human MAGED2 (NM_014599.6), SARS-CoV-2 Mpro WT, or C145A mutant coding sequences were constructed into pET28a vector separately. MAGED2 was with an N-terminal 2× Strep Tag. Mpro was tagged with an N-terminal GST and a C-terminal 6× His. 3C protease cleavage sites from Human Rhinovirus were added between Mpro and tags. The plasmids were transformed into *Escherichia coli* BL21 (DE3). When *E. coli* cultures were grown to a density (OD_600_) of 0.6 at 37°C, protein expression was induced with 0.5 mM final IPTG concentration, culturing at 16°C overnight. Next day, the bacteria were harvested and resuspended in lysis buffer (20 mM Tris-HCl, 300 mM NaCl, pH 8.0) and homogenized with an ultra-high-pressure cell disrupter at 4°C. The lysate was centrifugated at 14,000 rpm for 30 min to separate supernatant and pellet. MAGED2 and Mpro were purified by Strep-Tactin (Thermo Fisher Scientific) and Ni-NTA (QIAGEN) column affinity chromatography separately. The column was washed with lysis buffer five times. MAGED2 bound to the Strep-Tactin column was eluted with lysis buffer supplemented with 2.5 mM desthiobiotin. Mpro was eluted by cleavage buffer (50 mM Tris-HCl [pH 7.0], 150 mM NaCl) including 300 mM imidazole, and tags were removed by Human rhinovirus 3C protease digestion. Proteins were further purified by ion-exchange chromatography and size-exclusion chromatography. Proteins were stored −80°C for further use.

### *In vitro* polymerase activity assay

The *in vitro* enzymatic activity of SARS-CoV-2 polymerase complex was tested according to the previous study.

### MAGED2 *in vitro* cleavage assay

Purified MAGED2 was incubated with Mpro WT or C145A mutant in cutting buffer (50 mM Tris-HCl [pH 7.3], 1 mM EDTA) at 30°C for 2 h. After the incubation, the samples were analyzed by SDS-PAGE and stained with Coomassie blue.

### Molecular dynamics simulation

The conformation of the Mpro (WT, Beta, or Omicron)-MAGED2 complex was predicted using AlphaFold-Multimer (24), and the MAGED2 terminal residues without any secondary structure were deleted. All molecular dynamics simulations were performed by the GROMACS 2019.6 package (55), with the AMBER19SB-ILDN force field (56) and the TIP3P solvent model (57), where residue HIS132 in Mpro_Omicron set to HIP. The simulation system was added sufficient solvent and neutralized using 0.15 M NaCl. The simulation system will perform 5,000 steps of steepest descent energy minimization to eliminate unreasonable contacts, followed by 1 ns of NVT and 2 ns of NPT pre-equilibration with a time step of 2 fs. During the pre-equilibration, the protein structure was restrained with a force constant of 1,000 kJ·mol^−1^·nm^−2^. The temperature of the protein and non-protein was coupled to 300 K using the V-rescale thermostat (58), while the pressure for homogeneous conditions was coupled at 1 bar using the Berendsen barostats (59). Short-range electrostatic and van der Waals interactions were truncated at 1.0 nm, and long-range electrostatic interactions were calculated using the Particle Mesh Ewald algorithm (60). The neighbor list was updated every 20 steps with a cutoff of 1.2 nm. Finally, the pressure coupling algorithm was switched from Berendsen to Parrinello-Rahman (61) for the production simulations, which were run for 500 ns with a total of 5,000 frames saved. The simulated system stability was determined using the time-dependent root-mean-square deviation of the protein backbone structure. The last 100 ns (1,000 frames) of equalization trajectory was used to calculate relative binding-free energy between Mpro-MAGED2 using gmx_MMGBSA (62). Visualization of the protein structure used VMD (http://www.ks.uiuc.edu/Research/vmd/) and PyMol (https://github.com/schrodinger/pymol-open-source).

### Statistical analysis

Student’s *t* test or one-way analysis of variance with Tukey’s honestly significant difference test was used to test for statistical significance of the differences between the different group parameters. *P* values of <0.05 were considered statistically significant.

## SUPPLEMENTAL MATERIAL

Fig. S1 mbio.01373-23-s0001.tif Verification of the host proteins that could be potentially cleaved by SARS-CoV-2 Mpro.

Fig. S2 mbio.01373-23-s0002.tif Edman degradation method for sequencing the first five residues of MAGED2 cleavage product.

Fig. S3 mbio.01373-23-s0003.tif Molecular dynamics (MD) simulations of the Mpro-MAGED2 complex.

Fig. S4 mbio.01373-23-s0004.tif MAGED2 restricts SARS-CoV-2 infection.

Fig. S5 mbio.01373-23-s0005.tif MAGED2 is associated with SARS-CoV-2 nsp9, nsp12 and N protein.

Fig. S6 mbio.01373-23-s0006.tif MAGED2 does not affect SARS-CoV-2 core polymerase complex assembly and activity.

Fig. S7 mbio.01373-23-s0007.tif MAGED2 interacts with nucleocapsid protein through its N-terminal.

Supplemental legends mbio.01373-23-s0008.docx Legends for supplemental figures and tables.

Table S1 mbio.01373-23-s0009.xls Predictive nsp5 cleavage candidates.

Table S2 mbio.01373-23-s0010.xlsx Information of the primers and oligos.

ASM does not own the copyrights to Supplemental Material that may be linked to, or accessed through, an article. The authors have granted ASM a non-exclusive, world-wide license to publish the Supplemental Material files. Please contact the corresponding author directly for reuse.
