# Supplementary figures and images for: SARS-CoV-2 main protease cleaves MAGED2 to antagonize host antiviral defense
Source: mBio. 2023 Jul 13;14(4):e01373-23. doi: 10.1128/mbio.01373-23 (PMC10470497; doi:10.1128/mbio.01373-23)

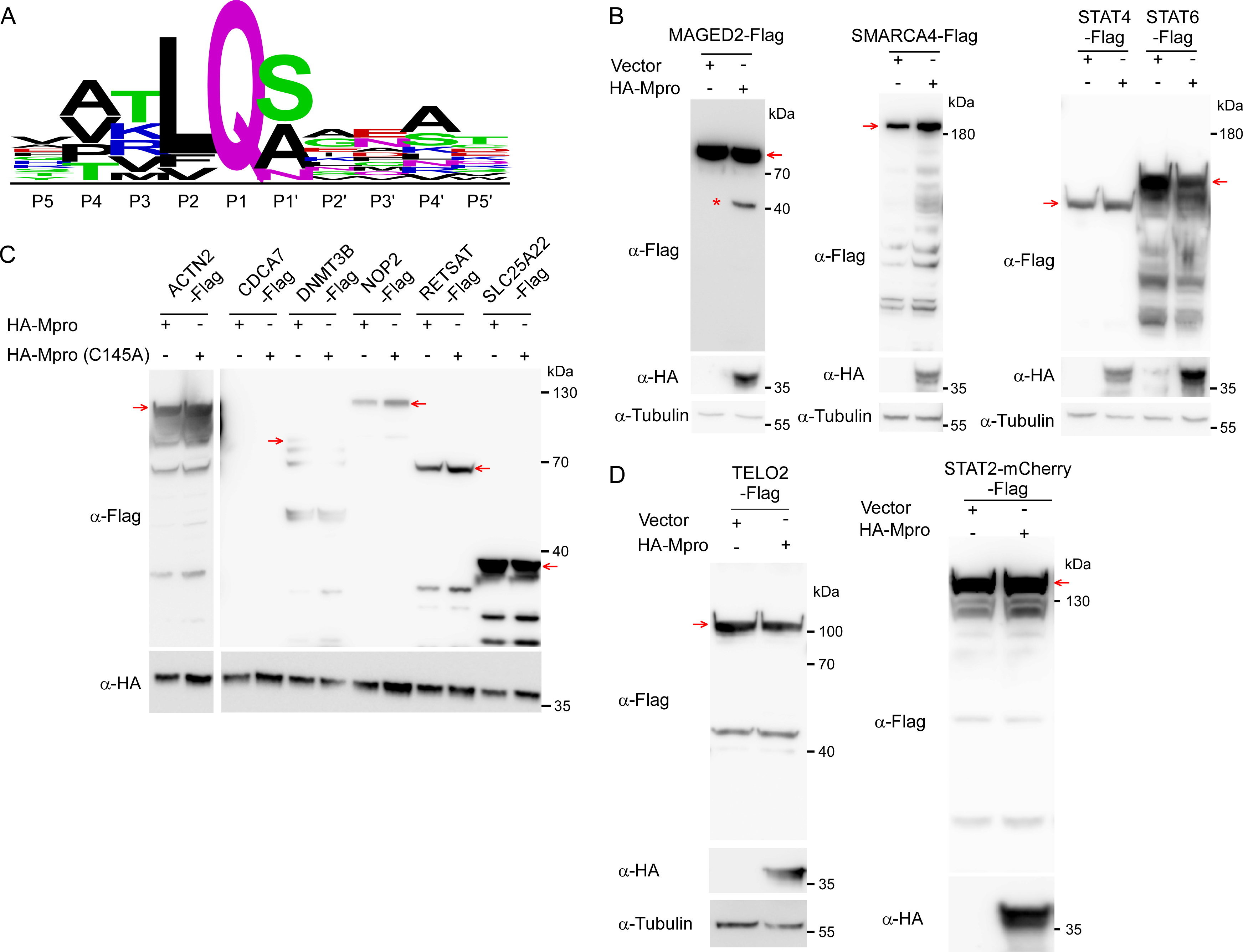

Supplement: Fig. S1 — Verification of the host proteins that could be potentially cleaved by SARS-CoV-2 Mpro. [file mbio.01373-23-s0001.tif]

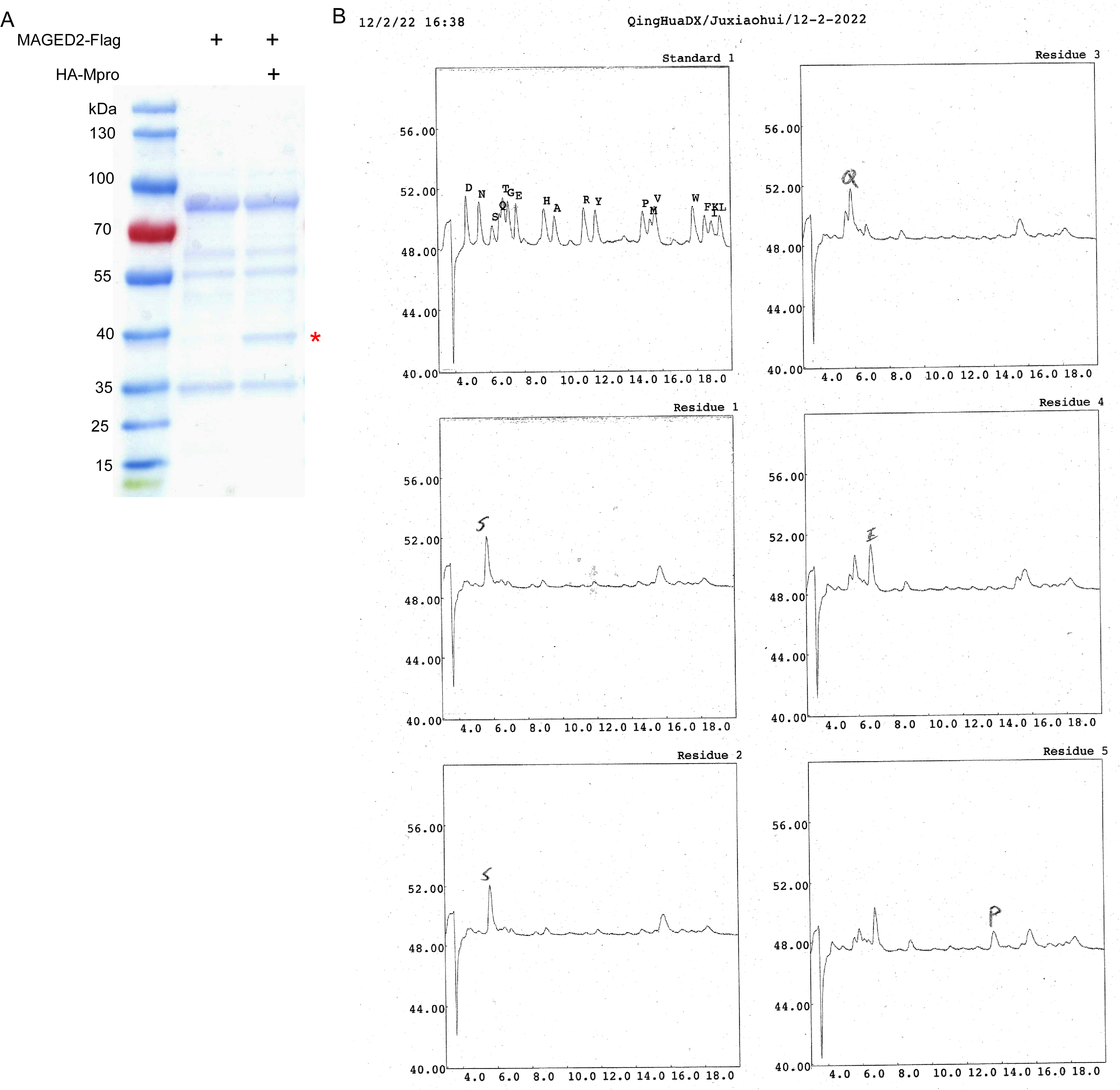

Supplement: Fig. S2 — Edman degradation method for sequencing the first five residues of MAGED2 cleavage product. [file mbio.01373-23-s0002.tif]

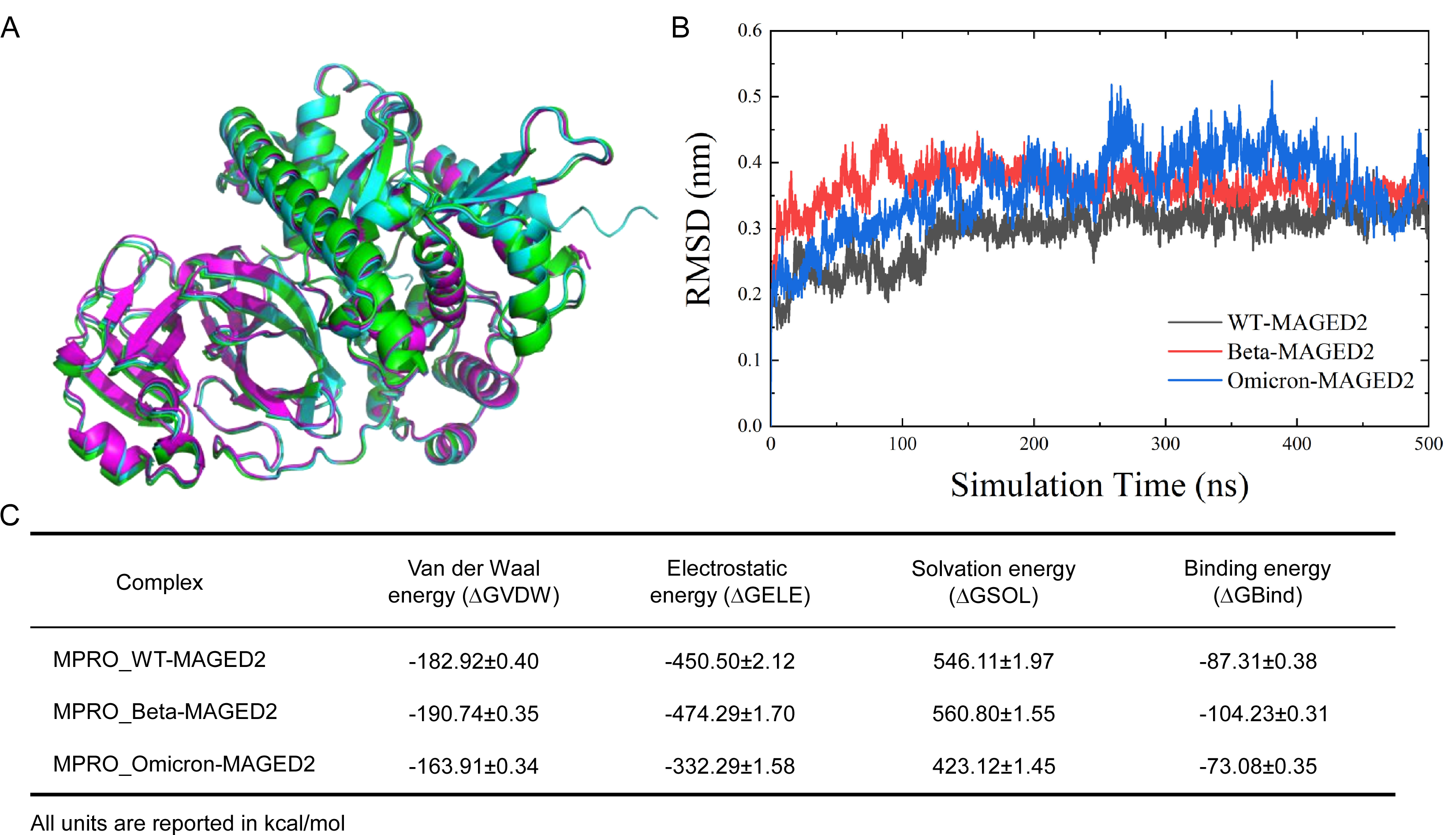

Supplement: Fig. S3 — Molecular dynamics (MD) simulations of the Mpro-MAGED2 complex. [file mbio.01373-23-s0003.tif]

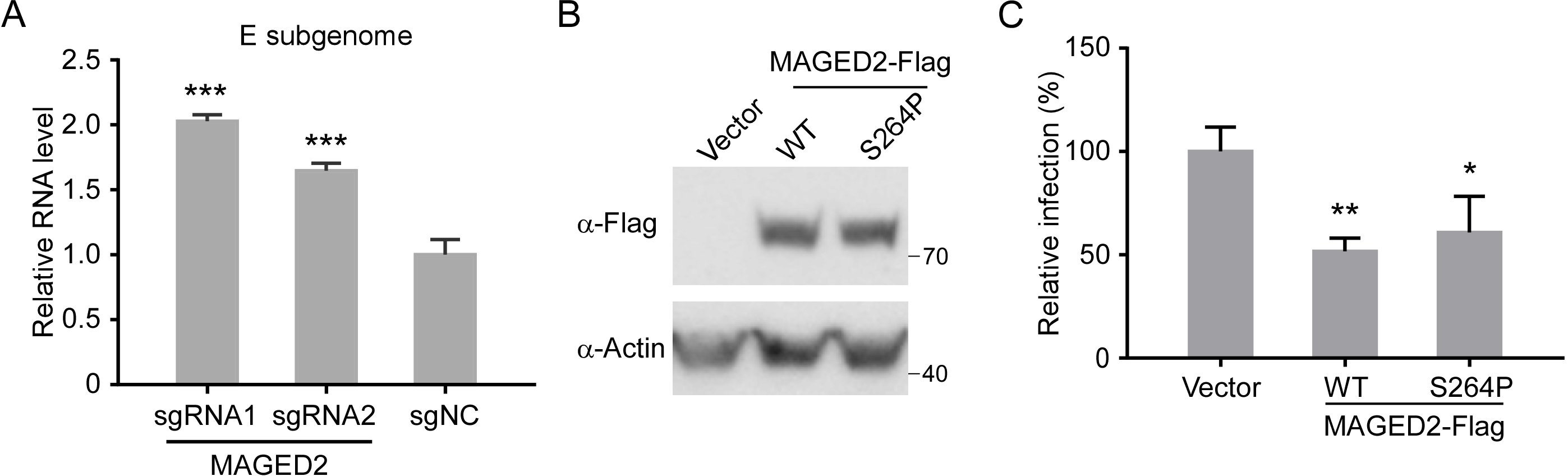

Supplement: Fig. S4 — MAGED2 restricts SARS-CoV-2 infection. [file mbio.01373-23-s0004.tif]

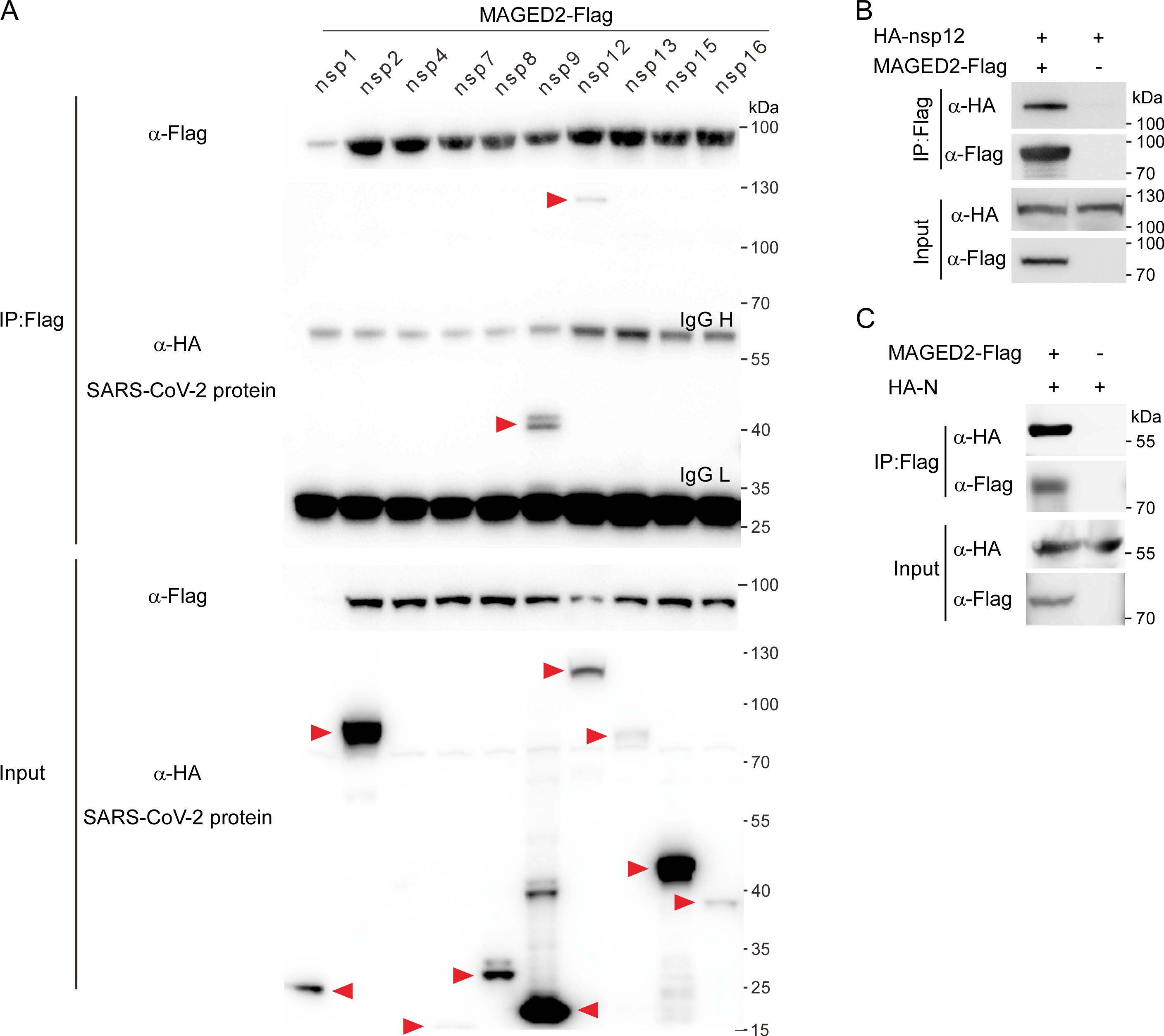

Supplement: Fig. S5 — MAGED2 is associated with SARS-CoV-2 nsp9, nsp12 and N protein. [file mbio.01373-23-s0005.tif]

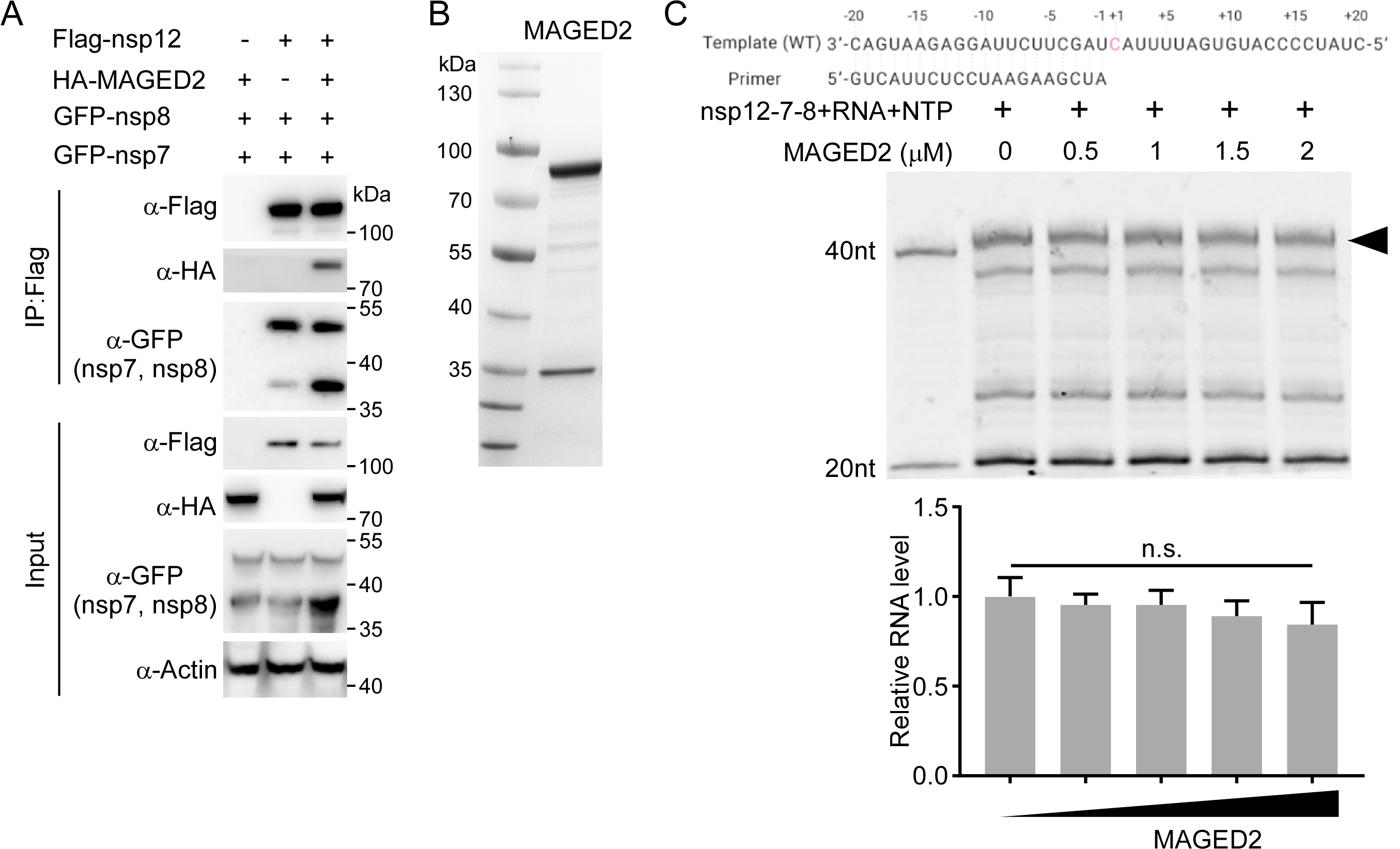

Supplement: Fig. S6 — MAGED2 does not affect SARS-CoV-2 core polymerase complex assembly and activity. [file mbio.01373-23-s0006.tif]

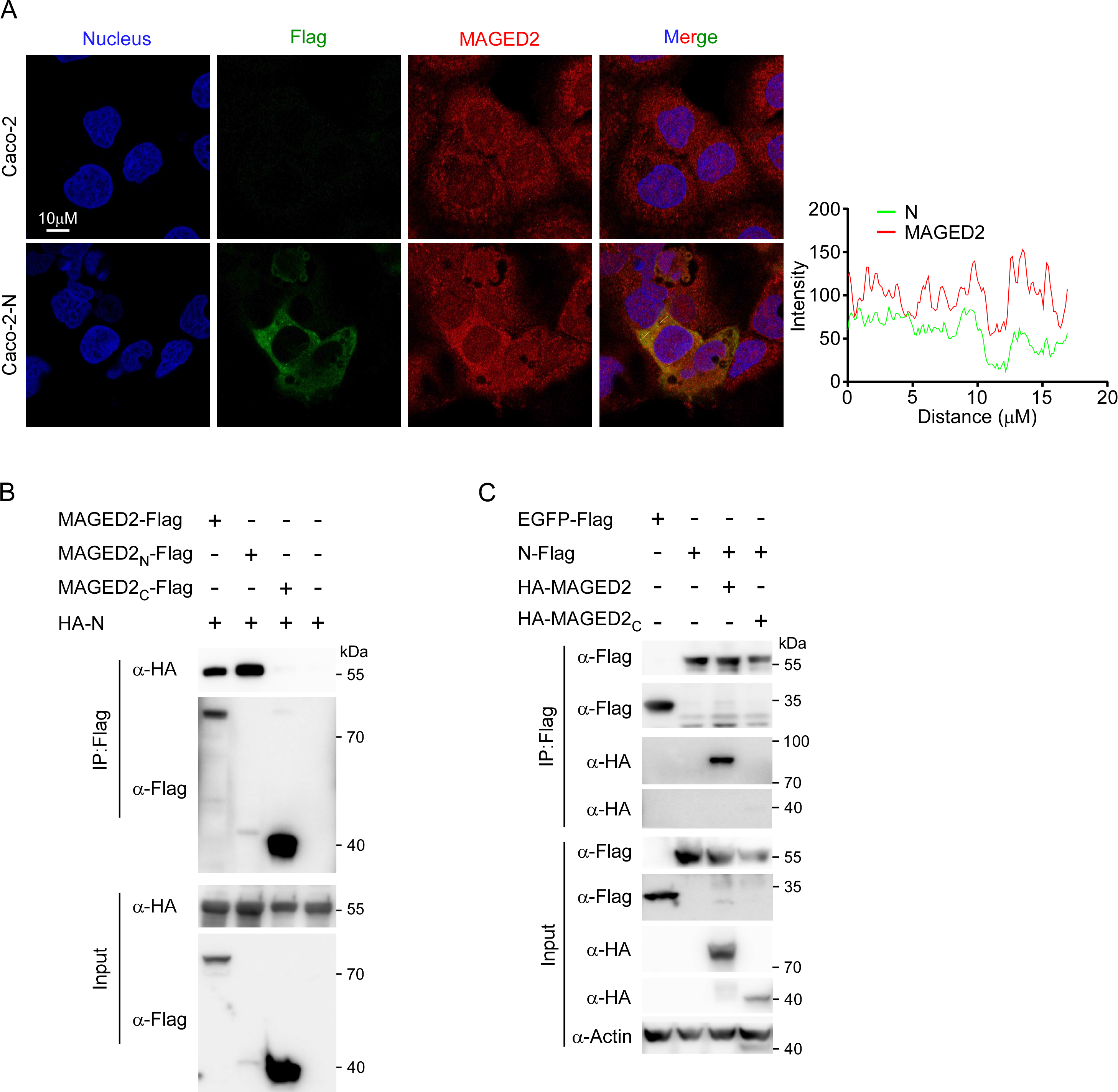

Supplement: Fig. S7 — MAGED2 interacts with nucleocapsid protein through its N-terminal. [file mbio.01373-23-s0007.tif]
