# Supplementary material for: SARS-CoV-2 main protease cleaves MAGED2 to antagonize host antiviral defense
Source: mBio. 2023 Jul 13;14(4):e01373-23. doi: 10.1128/mbio.01373-23 (PMC10470497; doi:10.1128/mbio.01373-23)
Supplement: Supplemental legends — Legends for supplemental figures and tables. [file mbio.01373-23-s0008.docx]

**Supplemental Figure legends**

**Figure S1. Verification of the host proteins that could be potentially cleaved by SARS-CoV-2 Mpro.** (**A**) Frequency distribution of amino acid residues surrounding Mpro cleavage sites at positions P5 to P5’ in nonstructural proteins was analyzed by WebLogo analysis (<https://weblogo.berkeley.edu/logo.cgi>). (**B-D**) Flag tagged host proteins as indicated were co-expressed with vector or HA-Mpro. Cell lysates were analyzed by western blot. Red arrow indicated expected full-length protein and red star indicated cleaved MAGED2.

**Figure S2. Edman degradation method for sequencing** **the first five residues of MAGED2 cleavage product**. (**A**) Flag tagged MAGED2 was co-expressed with vector or HA tagged Mpro in HEK293T cells. MAGED2 was immunoprecipitated by Flag antibody. Immunoprecipitants were subjected to SDS-PAGE and Coomassie blue staining in PVDF membrane. Red star indicated cleaved MAGED2 and cleaved MAGED2 was subjected to Edman sequencing. (**B**) Edman sequencing results of the first five residues of MAGED2 cleavage product (S-S-Q-E-P).

**Figure S3. Molecular dynamics (MD) simulations of the Mpro-MAGED2 complex.** (**A**) Conformation of the Mpro-MAGED2 complex predicted by AlphaFold-multimer. Wild-type Mpro (Green), Beta variant Mpro (Blue) and Omicron variant Mpro (Purple). Each Mpro corresponds to a MAGED2. (**B**) The time-evolution RMSD curves of Mpro (WT, Beta, or Omicron)-MAGED2 complex. Simulation time is 500 ns and three simulations were performed. (**C**) The relative binding free energy for the Mpro-MAGED2 complex. Smaller values of Binding energy indicate stronger bonding.

**Figure S4. MAGED2 restricts SARS-CoV-2 infection.** (**A**) Caco-2-N cells were infected with SARS-CoV-2 GFP/ΔN trVLP at a multiplicity of infection (MOI) of 0.1, and SARS-CoV-2 E subgenomic RNAs were analyzed by RT-qPCR at 24 hours post infection. (**B-C**) Human MAGED2 wild-type or S264P mutant were ectopically expressed in Caco-2-N cells by lentiviral transduction, and the cells were subsequently infected with SARS-CoV-2 GFP/ΔN trVLP at a multiplicity of infection (MOI) of 0.1. Cells were analyzed by flow cytometry at 24 hours post infection to determine the percentage of the trVLP infected cells. Values are means plus standard deviations (SD) (error bars) (n = 3). *, P < 0.05; **, P < 0.01; ***, P < 0.001 by one-way ANOVA.

**Figure S5. MAGED2 is associated with SARS-CoV-2 nsp9, nsp12 and N protein.** (**A-B**) Flag tagged MAGED2 and HA tagged SARS-CoV-2 nonstructural proteins were co-expressed in HEK293T cells. MAGED2 was immunoprecipitated by Flag antibody conjugated magnetic beads and the immunoprecipitants were blotted with Flag and HA antibodies. Red arrow indicated SARS-CoV-2 protein. (**C**) Flag tagged MAGED2 and HA tagged viral N protein were co-expressed in HEK293T cells. MAGED2 was immunoprecipitated by Flag antibody conjugated magnetic beads and the immunoprecipitants were blotted with Flag and HA antibodies. All data are representative of three independent experiments.

**Figure S6. MAGED2 does not affect SARS-CoV-2 core polymerase complex assembly and activity.** (**A**) Flag-nsp12, GFP tagged nsp7 and nsp8 were co-expressed in HEK293T cells. nsp12 was immunoprecipitated by Flag antibody conjugated with magnetic beads. Immunoprecipitants were analyzed with Flag, HA and GFP antibody. (**B**) Coomassie blue staining of purified MAGED2*.* (**C**) Comparison of RNA synthesis activities of the SARS-CoV-2 core polymerase complex in the presence of MAGED2. The first 40 nt template at the 3’ end of SARS-CoV-2 genome was annealed with complementary 20 nt primer containing a 5’-fluorescein label. Annealed RNA templates were added to the pre-mixed nsp12, nsp7, nsp8 with different amounts of MAGED2. The reaction was at 30 ℃ for 1 hour and stopped by heating at 95 ℃ for 10 min. Reaction substrates and products were resolved through 20% denaturing PAGE. Images were taken by a Vilber Fusion imaging system. Top band was fully elongated product and bottom was excess primer band. Intensity of RNA band was analyzed by ImageJ. n.s., not significantly different by one-way ANOVA. All data were representative of three independent experiments.

**Figure S7. MAGED2 interacts with nucleocapsid protein through its N-terminal.** (**A**) Immunofluorescence of N protein and MAGED2 in Caco-2 cells. Flag tagged N protein was expressed in Caco-2 cells by lentiviral transduction. Cells were stained with Flag (green) and MAGED2 (red) antibody prior to analysis by confocal microscopy. The cell nuclei were stained with DAPI (blue). Line profiles corresponding to the white dashed lines show colocalization. (**B**) HA-N protein and Flag tagged MAGED2 full-length or truncations were co-expressed in HEK293T cells. Cell lysate were immunoprecipitated by Flag antibody conjugated magnetic beads and immunoprecipitants were blotted by Flag and HA antibodies. (**C**) SARS-CoV-2 Gluc replicon RNA, plasmids encoding GFP-Flag or N-Flag and HA-MAGED2 were co-electroporated into HEK293T cells. Cells were collected at 24 hours post electroporation and GFP or N protein were immunoprecipitated by Flag antibody. The immunoprecipitants were blotted with Flag and HA antibodies. All data are representative of three independent experiments.

**Supplemental Tables**

Table S1. Predictive nsp5 cleavage candidates.

Table S2. Information of the primers and oligos.
